# Stopping renin-angiotensin system blockers after acute kidney injury and risk of adverse outcomes: parallel population-based cohort studies in English and Swedish routine care

**DOI:** 10.1186/s12916-020-01659-x

**Published:** 2020-07-29

**Authors:** Patrick Bidulka, Edouard L. Fu, Clémence Leyrat, Fotini Kalogirou, Katherine S. L. McAllister, Edward J. Kingdon, Kathryn E. Mansfield, Masao Iwagami, Liam Smeeth, Catherine M. Clase, Krishnan Bhaskaran, Merel van Diepen, Juan-Jesus Carrero, Dorothea Nitsch, Laurie A. Tomlinson

**Affiliations:** 1 Department of Non-Communicable Disease Epidemiology, London School of Hygiene and Tropical Medicine, Keppel Street, London, WC1E 7HT UK; 2 Department of Clinical Epidemiology, Leiden University Medical Center, Albinusdreef, Leiden, 2333ZA The Netherlands; 3 Cambridge University Hospitals NHS Foundation Trust, Cambridge Biomedical Campus, Hills Road, Cambridge, CB2 0QQ UK; 4 Sussex Kidney Unit, Royal Sussex County Hospital, Brighton, BN2 5BE UK; 5 Department of Health Services Research, Faculty of Medicine, University of Tsukuba, Ibaraki, Japan; 6 Department of Medicine, Department of Health Research, Evidence and Impact, McMaster University, Hamilton, ON Canada; 7 Department of Medical Epidemiology and Biostatistics, Karolinska Institutet, Nobels väg 12, Stockholm, Sweden

**Keywords:** Acute kidney injury, Heart failure, Angiotensin-converting enzyme inhibitor (ACEI), Angiotensin II receptor blocker (ARB)

## Abstract

**Background:**

The safety of restarting angiotensin-converting enzyme inhibitors (ACEI) or angiotensin II receptor blockers (ARB) after acute kidney injury (AKI) is unclear. There is concern that previous users do not restart ACEI/ARB despite ongoing indications. We sought to determine the risk of adverse events after an episode of AKI, comparing prior ACEI/ARB users who stop treatment to those who continue.

**Methods:**

We conducted two parallel cohort studies in English and Swedish primary and secondary care, 2006–2016. We used multivariable Cox regression to estimate hazard ratios (HR) for hospital admission with heart failure (primary analysis), AKI, stroke, or death within 2 years after hospital discharge following a first AKI episode. We compared risks of admission between people who stopped ACEI/ARB treatment to those who were prescribed ACEI/ARB within 30 days of AKI discharge. We undertook sensitivity analyses, including propensity score-matched samples, to explore the robustness of our results.

**Results:**

In England, we included 7303 people with AKI hospitalisation following recent ACEI/ARB therapy for the primary analysis. Four thousand three (55%) were classified as stopping ACEI/ARB based on no prescription within 30 days of discharge. In Sweden, we included 1790 people, of whom 1235 (69%) stopped treatment. In England, no differences were seen in subsequent risk of heart failure (HR 1.10; 95% confidence intervals (CI) 0.93–1.30), AKI (HR 0.90; 95% CI 0.77–1.05), or stroke (HR 0.99; 95% CI 0.71–1.38), but there was an increased risk of death (HR 1.27; 95% CI 1.15–1.41) in those who stopped ACEI/ARB compared to those who continued. Results were similar in Sweden: no differences were seen in risk of heart failure (HR 0.91; 95% CI 0.73–1.13) or AKI (HR 0.81; 95% CI 0.54–1.21). However, no increased risk of death was seen (HR 0.94; 95% CI 0.78–1.13) and stroke was less common in people who stopped ACEI/ARB (HR 0.56; 95% CI 0.34–0.93). Results were similar across all sensitivity analyses.

**Conclusions:**

Previous ACEI/ARB users who continued treatment after an episode of AKI did not have an increased risk of heart failure or subsequent AKI compared to those who stopped the drugs.

## Background

Acute kidney injury (AKI) is a sudden (over hours or days) deterioration in kidney function, strongly associated with a range of adverse outcomes, particularly an increase in subsequent admission with heart failure [1, 2]. Risk factors for AKI include diabetes, chronic kidney disease, hypertension, and heart failure with reduced ejection fraction (HFrEF) [3]. ACE inhibitors (ACEI) and angiotensin II receptor blockers (ARB) are commonly used, evidence-based treatments for these conditions, and thus, many people admitted to hospital with AKI are treated with these medications. It is widely believed that in the setting of acute intercurrent illness, ACEI/ARB can cause or exacerbate AKI. Internationally, strategies have been developed to improve outcomes from AKI, and among these are guidance to temporarily reduce or stop ACEI/ARB in acutely unwell people with the aim of preventing or reducing the severity of AKI [4–6]. However, there is concern that people who have medication stopped during acute illness may not have it restarted, which may result in adverse outcomes, particularly decompensation for those with HFrEF [3].

Evidence about the comparative risks of cardiovascular and kidney outcomes for people who have ACEI/ARB medications stopped after an episode of AKI is limited. The lack of clear guidance about whether, and when, to restart treatment suggests a degree of variation in care regarding post-AKI exposure to ACEI/ARB; in this situation, observational research may provide valuable insights.

Therefore, we sought to examine prescribing of ACEI/ARB after a first episode of AKI and determine the risks of subsequent hospital admission with heart failure, further AKI, or stroke, and the risk of death, comparing people who stopped treatment with those where ACEI/ARB was continued in primary care after hospital discharge. To ensure the robustness of our results, we undertook this analysis in England and Sweden to compare effects in different data sources, with different primary health care systems and differing recognition and coding of AKI.

## Methods

### Study design and setting

#### English cohort

This was a population-based cohort using linked data from the UK Clinical Practice Research Datalink (CPRD-GOLD) and Hospital Episode Statistics (HES) databases. CPRD-GOLD contains primary care data for approximately 7% of the current UK population and is largely representative of that population in terms of age, sex, and ethnicity [7]. It contains data on clinical symptoms and diagnoses recorded using Read codes and prescription data. HES records include all hospital admission data for National Health Service (NHS)-funded patients in England. We used only CPRD-HES linked data, which is available for approximately 60% of general practices contributing to CPRD and limits the analysis to people contributing to CPRD from England [8]. The study period was 1 January 2010 to 31 December 2016. We restricted the study period in the UK to more recent years because awareness and coding of AKI has markedly increased since 2010 [9].

#### Swedish cohort

The Swedish cohort used data from the Stockholm CREAtinine Measurements (SCREAM) healthcare utilisation cohort, which includes all Stockholm residents (Sweden) accessing healthcare with at least one plasma creatinine measured during 2006–2011 [10, 11]. SCREAM includes data from ~ 1.3 million adults, corresponding to 68% of the region for the study period. Laboratory results were linked with regional and national administrative databases for complete information on demographic data, healthcare use (both inpatient and outpatient care), diagnoses, dispensed drugs at Swedish pharmacies, and death.

### Participants

#### English cohort

Study participants were aged 18 years or over and met criteria for research-standard data during the study period, with at least 1 year of continuous registration before the study entry to ensure reliable measures of drug use and baseline covariates. Participants were included at the first recorded hospital admission with AKI defined using International Classification of Diseases (ICD)-10 code N17 in the first or second diagnostic position in HES data in any episode within 1 week of admission. The N17 code has a positive predictive value for AKI of 95% in an English single-centre study [12]. We then restricted to participants prescribed ACEI/ARB within 60 days before the AKI admission. We excluded people with a history of AKI admission before 2010, end-stage renal disease (ESRD) in primary care records, or dialysis procedure codes in secondary care records before the AKI admission.

#### Swedish cohort

We defined participants included in the Swedish cohort in the same way as in England, except that we identified those included based on a dispensed prescription for ACEI/ARB within 180 days before the AKI admission (rather than 60 days in England) because of the longer duration of prescriptions in Sweden. We excluded people admitted for AKI before 2006 or with a history of previous dialysis or transplant.

### Exposure

We compared people who stopped ACEI/ARB therapy to those who continued the drugs. In the primary analysis, individuals stopping therapy were defined as those with no record of being prescribed an ACEI/ARB in primary care (English cohort) or dispensed an ACEI/ARB from a pharmacy (Swedish cohort) within 30 days following the baseline AKI discharge. Individuals continuing therapy were defined as those who were prescribed or dispensed an ACEI/ARB within the same period. The 30-day period was chosen to allow for medication prescribed on discharge, stockpiled medication, and delays in attending their primary care doctor to arrange a repeat prescription. Inspection of the data confirmed that most ACEI/ARB prescriptions occurred within 30 days of discharge.

### Outcomes

The primary outcome was a hospital admission with heart failure, defined by ICD-10 coding in the first or second diagnostic position of the first episode in HES inpatient data (English cohort) or hospital discharge records (Swedish cohort) during the follow-up period. Secondary outcomes were hospital admission for AKI and stroke (similarly defined) and death from all causes. For the Swedish cohort, ICD-10 codes used to define outcomes were based on validated algorithms in the Swedish health care setting [13]. To avoid immortal time bias, follow-up for all participants began 30 days post-AKI discharge; after this time, the exposure group could be definitively assigned to all participants. We excluded people who ended follow-up in the database (e.g. moved to a non-participating GP (English cohort), migrated out of the region of Stockholm (Swedish cohort)), died, or had the outcome of interest during the first 30 days after AKI discharge. The latter exclusion was outcome-specific, so a participant readmitted with heart failure within the first 30 days would be excluded from the heart-failure-outcome analysis post-30 days, but remained eligible for inclusion in analyses of other outcomes (AKI, stroke, death). During included follow-up (≥ 30 days from discharge), participants having one type of outcome were similarly considered to remain at risk for other outcomes after that date. We followed participants for a maximum of 2 years to minimise the influence on outcomes of later events and changes in prescribing. The study design is shown graphically in Additional file 1: Figure S1.

### Covariates

#### English cohort

We chose covariates for multivariable model adjustment and propensity score matching a priori, based on previous knowledge. We included age, sex, smoking, alcohol intake, body mass index (BMI) [14], and calendar year (to account for the changes in practice that have influenced drug prescribing and coding of hospital outcomes). We also considered other confounders including baseline comorbidities (arrhythmia, diabetes, heart failure, hypertension, ischaemic heart disease) defined using morbidity coding in primary care (Read codes) or as part of a hospital admission (ICD-10 codes) any time prior to the baseline AKI admission. Heart failure was a critical baseline comorbidity that may be incompletely captured by Read codes in CPRD [15], so this was additionally defined by a prescription for spironolactone within 60 days before baseline AKI admission [15]. We adjusted for baseline kidney function by calculating the estimated glomerular filtration rate (eGFR) using the most recent serum creatinine recorded in participants’ primary care records (excluding any measures within 7 days before baseline AKI admission) and using the CKD-EPI equation, assuming non-black ethnic origin for all [16]. eGFR was categorised using the cut-points of the Kidney Disease Improving Global Outcomes guidelines for chronic kidney disease (CKD), but without the requirement for two measures 3 months apart [17]. Where eGFR was missing, we assigned a ‘no known CKD’ category as in this population missing serum creatinine data is associated with outcomes closely equivalent to normal kidney function [18]. We also adjusted for concomitant prescriptions for loop diuretics, beta-blockers, and calcium channel blockers, defined by a prescription within the 60 days before the baseline AKI admission.

#### Swedish cohort

We adjusted for similar covariates to those used in England, except that we defined all variables at 30 days post AKI discharge (the inception point for the cohort). We could not capture BMI, ethnicity, smoking, or alcohol intake in Swedish data. Spironolactone use was adjusted for as a concomitant medication.

### Analysis

In both the English and Swedish cohorts, after confirming the proportional hazard assumption was met, we used Cox regression to estimate adjusted hazard ratios (HR) for admission with heart failure (primary analysis), AKI, stroke, and death, comparing participants who stopped treatment with those who were prescribed (England) or dispensed (Sweden) an ACEI/ARB within 30 days after discharge. Given the low level of missing data (8% of English participants for one or more covariates, none in the Swedish cohort) and that data were unlikely to be missing at random, we used a complete-case approach. In England, analysis was conducted using Stata version 15.1 (StataCorp, Texas) and R version 3.5.0, and in Sweden, R version 3.4.2.

### Sensitivity analyses

To maximise comparability between our exposure groups, we conducted propensity-score-matched analyses to estimate the HRs for each study outcome. We used logistic regression to calculate the odds of stopping or being prescribed ACEI/ARB by the start of follow-up for each year, adjusting for relevant covariates. Factors included in the propensity score model were all covariates described previously; plus in the English cohort, we also adjusted for frailty using the electronic frailty index (eFI) as a covariate, including both the frailty category (fit, mildly, moderately or severely frail) and the individual components comprising the score [19]. We did not adjust for eFI in the primary analysis to maintain comparability with the Swedish cohort where these data were not available. Participants who stopped ACEI/ARB were matched 1:1 on propensity score to participants who continued therapy, using nearest neighbour matching with a calliper of 0.2. We measured standardised mean differences (SMD) for each covariate to check for balance between groups, with an SMD < 10% indicating acceptable balance. We estimated HR using Cox regression for admission with heart failure, AKI, and stroke and for death comparing those who stopped ACEI/ARB with those who continued the drugs.

In addition, in both cohorts unless specified, we conducted further sensitivity analyses. Firstly, we defined people as having stopped ACEI/ARB if they had not received an ACEI/ARB prescription in primary care by 60 days after discharge following their AKI admission (rather than 30 days in the main analysis). Secondly, to increase certainty that participants were regular users of ACEI/ARB, we restricted the cohort to people who had at least two prescriptions for ACEI/ARB within 60 days before the baseline AKI admission, one of which was within 30 days before admission (England only). Thirdly, we restricted outcome events to those recorded in the first diagnostic position in English or Swedish hospital records only (in the main analysis, we used the first and second diagnostic positions). Fourth, we determined HRs for all outcomes after excluding people who required dialysis during their initial AKI admission (England only). Fifth, we conducted stratified analysis in three time intervals (0–29, 30–89, and 90+ days after start of follow-up) to consider changes over time in the risk of re-admission and death in participants who stopped ACEI/ARB compared with those who continued. Finally, we repeated all analyses excluding people who were readmitted to hospital in the first 30 days after the index AKI discharge.

### Patient involvement

No patients were involved in setting the research question or the outcome measures, nor were they involved in developing plans for design or implementation of the study. No patients were asked to advise on interpretation or writing up of results. We are not able to disseminate the results of the research directly to study participants because the data used in both cohorts were de-identified.

## Results

### Study populations and baseline characteristics

**Fig. 1:**
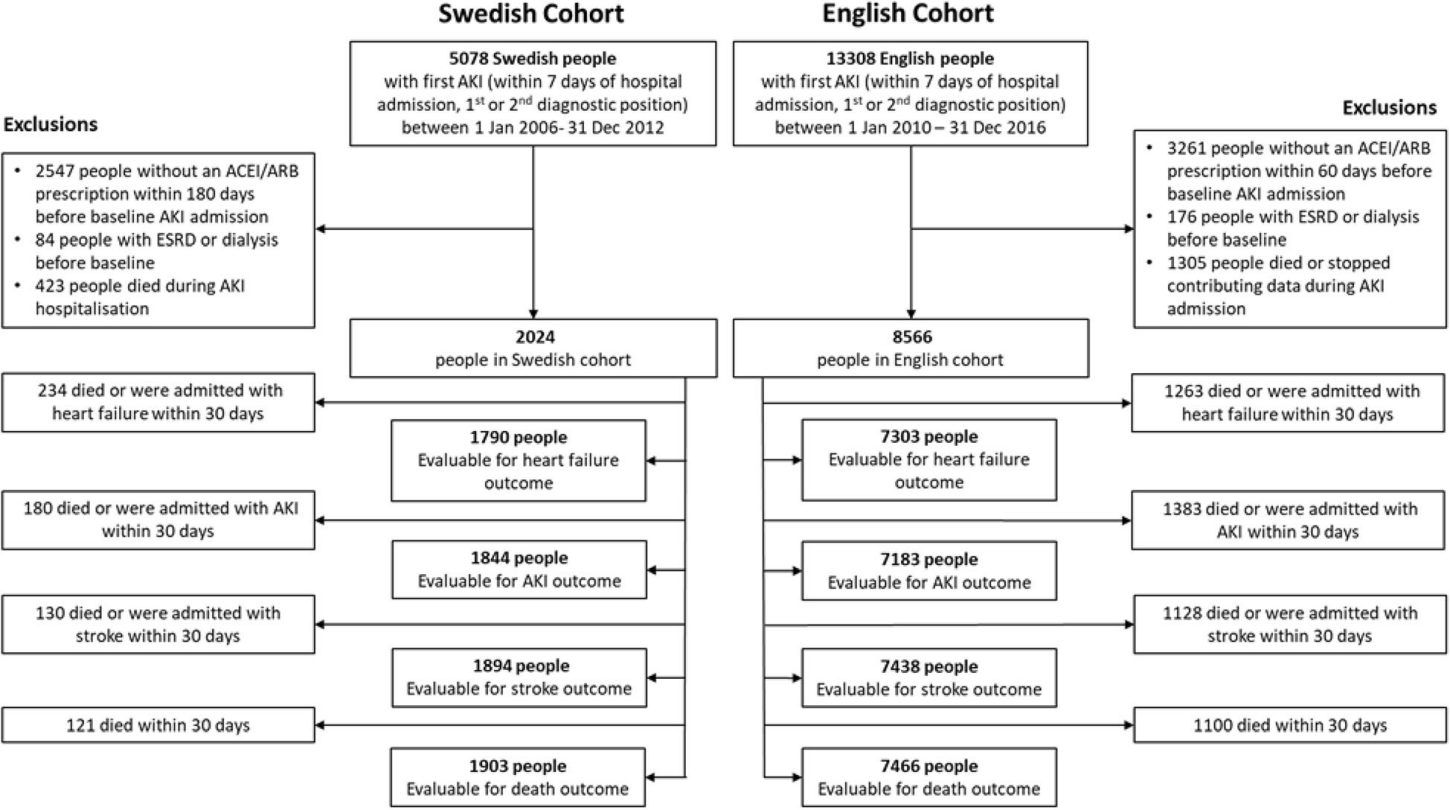
Flow diagram for study participant identification

We included 8566 people in the English cohort and 2024 in the Swedish cohort (Fig. 1). Total follow-up time for the primary outcome (heart failure) was 8215 person-years in the English cohort and 2445 person-years in the Swedish cohort. In the English cohort, AKI was listed in the 1st diagnostic position in hospital data for 38% of participants (Additional file 1: Table S1). For 62%, AKI was listed in the 2nd diagnostic position, and for these people, infections were the most common group of primary diagnoses.

Baseline characteristics for the English and Swedish cohorts in the heart-failure-outcome analysis, stratified by stopping or continuing ACEI/ARB, are shown in Table 1. Comparisons between the English and Swedish cohorts for all participants discharged after AKI and those included in the heart-failure-outcome analyses are shown in Additional file 1: Table S2. The cohorts for the primary analysis were similar between countries with a mean age of 77 years and 75 years in England and Sweden respectively, 53% and 55% men, and similar mean baseline eGFR 54 mL/min/1.73 m^2^ (standard deviation [SD] ~ 23). Slightly more participants in the English cohort were diabetic (47%) compared with the Swedish cohort (43%). A lower proportion of the English cohort was classified as having heart failure (30%) compared with the Swedish cohort (47%).

**Table 1:** Baseline characteristics of the English and Swedish cohorts included in the heart failure (primary outcome) analysis

	English cohort		Swedish cohort	
	Continued ACEI/ARB	Stopped ACEI/ARB	Continued ACEI/ARB	Stopped ACEI/ARB
	*N* = 3300	*N* = 4003	*N* = 555	*N* = 1235
**Age (years), mean (SD)**	77 (11)	77 (11)	75 (12)	75 (12)
**Age (years)**				
18–69	756 (23)	914 (23)	179 (32)	393 (32)
70–74	386 (12)	482 (12)	73 (13)	162 (13)
75–79	609 (19)	731 (18)	80 (14)	161 (13)
80–84	672 (20)	827 (21)	94 (17)	214 (17)
85–89	552 (17)	655 (16)	85 (15)	201 (16)
≥ 90	325 (10)	394 (10)	44 (8)	104 (8)
**Women**	1569 (48)	1862 (47)	255 (46)	550 (45)
**Baseline eGFR (ml/min/1.73 m**^**2**^**), mean (SD)**	55 (21)	53 (22)	53 (23)	55 (24)
**eGFR category**				
No known CKD	388 (12)	487 (12)	25 (5)	102 (8)
G1-No CKD	202 (6)	214 (5)	38 (7)	106 (9)
G2-Mild	894 (27)	999 (25)	164 (30)	357 (29)
G3a-Mild-Mod	726 (22)	838 (21)	118 (21)	237 (19)
G3b-Mod-Severe	775 (24)	911 (23)	112 (20)	242 (20)
G4-Severe	297 (9)	504 (13)	83 (15)	153 (12)
G5-Kidney failure	18 (1)	50 (1)	15 (3)	38 (3)
**Comorbidities**				
Arrhythmia	1069 (32)	1180 (30)	233 (42)	452 (37)
Diabetes	1596 (48)	1824 (46)	259 (47)	507 (41)
Heart failure	1067 (32)	1146 (29)	285 (51)	563 (46)
Hypertension	2869 (87)	3470 (87)	472 (85)	1078 (87)
IHD	1770 (54)	1981 (50)	213 (38)	445 (36)
**Medications**				
Beta blocker	1453 (44)	1643 (41)	389 (70)	825 (67)
CCB	1152 (35)	1458 (36)	205 (37)	452 (37)
Loop diuretic	1942 (59)	2273 (57)	168 (30)	331 (27)
Spironolactone	416 (13)	432 (11)	144 (26)	279 (23)
**Ethnicity**				
White	3058 (93)	3812 (95)		
Black	59 (2)	45 (1)		
Asian	119 (4)	85 (2)		
Other	35 (1)	26 (1)		
Missing	29 (1)	35 (1)		
**Smoking**				
Non-smoker	986 (30)	1211 (30)		
Ex-smoker	1923 (58)	2360 (59)		
Current smoker	378 (12)	425 (11)		
Missing	13 (< 1)	7 (< 1)		
**Alcohol use**				
Non-drinker	389 (12)	433 (11)		
Current drinker	2133 (65)	2683 (67)		
Ex-drinker	611 (19)	713 (18)		
Missing	167 (5)	174 (4)		
**BMI (kg/m**^**2**^**), mean (SD)**	29 (7)	29 (7)		
**BMI (kg/m**^**2**^**)**				
BMI < 18.5	84 (3)	83 (2)		
BMI 18.5–24.9	770 (23)	1019 (25)		
BMI 25–29.9	1061 (32)	1278 (32)		
BMI ≥ 30	1265 (38)	1484 (37)		
Missing	120 (4)	139 (4)		
**Dialysis during baseline AKI admission**	71 (2)	190 (5)		

Data are n (%) unless otherwise specified. BMI, alcohol use, smoking, and ethnicity were not available in the Swedish dataset

*SD* standard deviation, *CKD* chronic kidney disease, *eGFR* estimated glomerular filtration rate, *eGFR category* using KDIGO cut-points without the chronicity requirement, *mod* moderate, *IHD* ischaemic heart disease, *CCB* calcium channel blockers, *BMI* body mass index, *AKI* acute kidney injury

### ACEI/ARB prescriptions

In England, of all people discharged from hospital following an admission with AKI, 45% (3833/8566) had not been prescribed ACEI/ARB by 90 days after hospital discharge (Additional file 1: Figure S2A). Of the people who did continue ACEI/ARB, 75% (3565/4733) had been prescribed the drugs by 30 days after discharge and 93% (4402/4733) at 60 days. In the heart-failure-outcome analysis, 55% (4003/7303) were classified as stopping ACEI/ARB based on no issued prescription within 30 days of discharge. The proportion of people prescribed ACEI/ARB after discharge in England increased progressively from 2010 (Additional file 1: Figure S3).

In the Swedish cohort, a higher proportion of people (53% (1083/2024)) had not been dispensed a prescription for ACEI/ARB from their pharmacy at 90 days post-discharge after admission with AKI (Additional file 1: Figure S2B). Of the people who did continue ACEI/ARB treatment, 67% (628/941) were dispensed a prescription within 30 days after discharge and 82% (771/941) at 60 days. In the heart-failure-outcome analysis, 69% (1235/1790) were classified as stopping ACEI/ARB based on no dispensed prescription within 30 days of discharge.

Characteristics of people who stopped or continued treatment were generally similar, although in the English cohort category G4 and G5 eGFR tended to be more common in those who stopped ACEI/ARB compared with those who continued (14% compared with 10%, respectively), as was dialysis during admission (5% vs 2%). In both cohorts, participants were slightly more likely to continue ACEI/ARB after discharge with AKI if they had baseline heart failure (32% vs 29% in England and 51% vs 46% in Sweden), diabetes (48% vs 46% in England and 47% vs 41% in Sweden), or ischaemic heart disease (54% vs 50% in England and 38% vs 36% in Sweden).

### Admissions

Rates of admissions for all outcomes were highest during the 30 days after discharge from the baseline AKI event (participants who were censored due to death or admission to hospital during this period are described in Additional file 1: Table S3). Compared with those who were included in the outcome analyses, people excluded in the 30 days after discharge were older and more likely to be women, with a higher prevalence of comorbidities including heart failure and lower baseline eGFR (Additional file 1: Table S3).

### Association of stopping ACEI/ARB with outcomes

In the English cohort, from 30 days after discharge, during a median follow-up of 1.1 years (interquartile range (IQR) 0.4–2.0), 611/7303 (8%) participants experienced a heart failure outcome, 716/7183 (10%) had AKI, 164/7438 (2%) a stroke, and 1885/7466 (25%) died. In the Swedish cohort, again from 30 days after discharge, during a median follow-up of 1.4 years (IQR 0.4–2.0), 345/1790 (19%) participants experienced a heart failure outcome, 102/1844 (6%) had AKI, 64/1894 (3%) a stroke, and 516/1903 (27%) died (Additional file 1: Table S4).

**Fig. 2:**
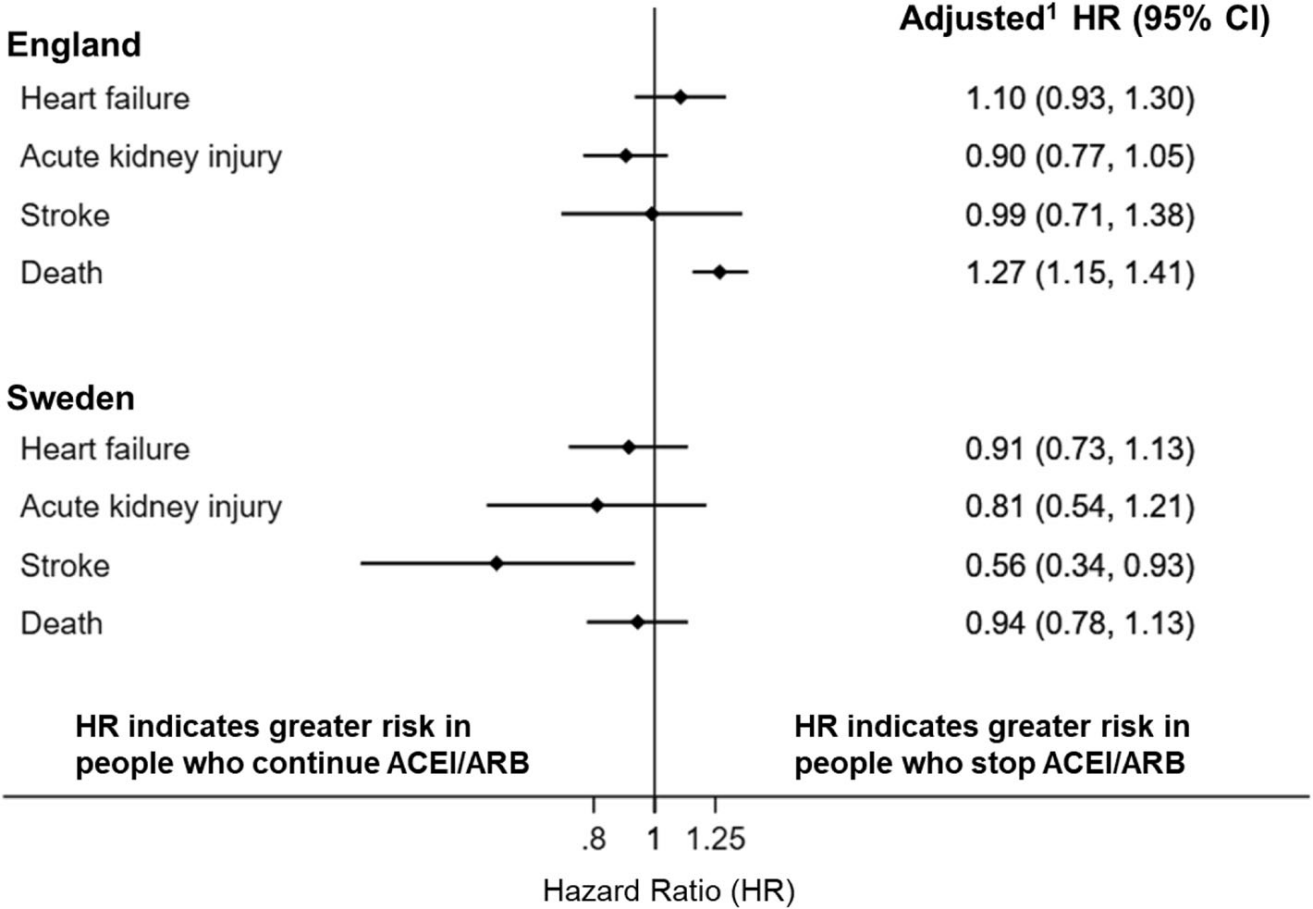
Forest plot of fully adjusted hazard ratios comparing people who stopped ACEI/ARB after an admission with AKI to those who continued, in English and Swedish cohorts, for each of the outcomes studied. Superscript number 1 indicates *English models* adjusted for age, sex, ethnicity, smoking, alcohol, BMI, eGFR category, diabetes, arrhythmia, heart failure, hypertension, ischaemic heart disease, beta-blocker, calcium channel blocker, diuretics, and discharge year. *Swedish models* adjusted for age, sex, eGFR category, diabetes, arrhythmia, heart failure, hypertension, ischaemic heart disease, beta-blocker, calcium channel blocker, diuretics, spironolactone, and discharge year

The fully adjusted hazard ratios (HR) for heart failure (primary outcome), AKI, stroke, and death in the English and Swedish cohorts are shown in Fig. 2 and Additional file 1: Table S4. The full model for heart failure in both cohorts, showing different levels of covariate adjustment, is presented in Additional file 1: Table S5. There was no difference in risk of admission with heart failure or AKI in either cohort between those who stopped or continued ACEI/ARB after discharge. Additionally, in the English cohort, there was no difference in the risk of stroke (HR 0.99, 95% CI 0.71–1.38) while in Sweden a lower risk of stroke was seen in those who stopped ACEI/ARB (HR 0.56, 95% CI 0.34–0.93). In the English cohort, but not in the Swedish cohort, we observed an increased risk of death in those who stopped ACEI/ARB compared with those who continued (England: HR 1.27, 95% CI 1.15–1.41; Sweden: 0.94, 95% CI 0.78–1.13).

### Sensitivity analyses

Propensity score (PS) distribution between exposure groups before and after PS matching in the English and Swedish cohorts (bottom figures) are shown in Additional file 1*:* Figure S4. Baseline characteristics of exposure groups for the heart failure outcome analysis after propensity score (PS) matching and standardised mean differences (SMD) before and after PS matching in the English and Swedish cohorts are shown in Additional file 1: Table S6.

We did not see any important differences from the main results in the propensity-score-matched analyses or sensitivity analyses by differing definitions of stopping, by different outcome definitions, or by time period post discharge for the outcomes heart failure, AKI, or stroke (Additional file 1: Figure S5 and Tables S7–10). Results were suggestive that the association between stopping ACEI/ARB and death in the English cohort became less marked at later time periods after start of follow-up (HR 1.59 (95% CI 1.21–2.08) at 0–29 days, 1.41 (95% CI 1.12–1.76) at 30–89 days, and 1.18 (95% CI 1.05–1.33) at more than 90 days (Additional file 1: Table S10)).

## Discussion

In both England and Sweden, we found no evidence of an increased rate of admissions for heart failure, AKI, or stroke among people who stopped ACEI/ARB treatment following discharge after AKI hospitalisation compared with people who continued, consistent across all main and sensitivity analyses. In Sweden, there was a lower risk of stroke among people who stopped ACEI/ARB although the numbers of stroke events were low and should be interpreted with caution. In England, but not in Sweden, there was an increased risk of death among people who stopped ACEI/ARB.

Our aim was to address an important clinical question regarding the possible harms and benefits of continuing or restarting ACEI/ARB after an episode of AKI. We showed consistent results for heart failure and AKI outcomes in two well-defined cohorts that contain granular detail about drug prescribing across two countries with different health care systems. This strengthens our findings and increases the generalisability of these results. People who continued ACEI/ARB and those who stopped were largely similar in terms of observed baseline characteristics, and a propensity-score analysis including detailed adjustment for frailty, a potential residual confounder, supports our findings.

However, there are also limitations to our study, which we sought to address through our study design and sensitivity analyses. The most important is that there are possible unmeasured differences between the groups leading to residual confounding. These include the original indication for drug prescription and, in particular, the presence and severity of left ventricular dysfunction.

We identified AKI using ICD-10 codes which, in England, are known to have a high positive predictive value for AKI, but also a high false-negative rate [12]. This means that the people included in the cohort are likely to be true cases for their baseline admission, although we may have missed less severe AKI admissions. The high false-negative rate could also have led to differential misclassification of the AKI outcome if those who continued ACEI/ARB were more likely to have an admission coded as AKI, or AKI coded in a higher diagnostic position, compared to those who stopped. This would have biased our effect estimate away from the null. Nonetheless, our results suggest no change in risk between exposure groups for subsequent AKI, and results were robust to changing the definition of the AKI outcome by coding position.

Lack of information about measures of creatinine in hospital meant that we could not grade severity of AKI according to the KDIGO criteria [20]; however, a sensitivity analysis excluding people who required dialysis during their baseline admission did not alter our results. We were also unable to ascertain whether the cause of AKI or misclassification of participants who actually had progressive chronic kidney disease influenced our results. We chose a priori not to adjust for creatinine or potassium levels after discharge in our analysis, as we thought this would be differentially missing for the sickest people who were readmitted before they could have outpatient blood tests, and blood test results may be on the causal pathway of the decision to restart ACEI/ARB. However, where available to clinicians, levels of kidney function and potassium are likely to influence practice in routine care.

In defining ACEI/ARB exposure, we did not have data for in-hospital treatment or discharge prescribing. We chose our 30-day post-discharge period to define exposure using primary care prescriptions partly because participants in England would have been likely to receive medication ‘to take away’ from hospital. Therefore, people classified as ‘continuers’ in our analysis could experience a range of treatment patterns, such as continuing ACEI/ARB without a break and stopping the drugs for a period but receiving a prescription within 30 days post AKI discharge. It is also possible that people did not take the medication as prescribed or that they were given specific instructions about whether, or when, to restart ACEI/ARB drugs. These issues could all have led to exposure misclassification which may have influenced the risk of subsequent outcomes in ways not captured by our analysis. Exposure to ACEI/ARB exposure would be misclassified if people were prescribed the medications after 30 days. However, the vast majority of prescriptions occurred within 60 days post-discharge, and our sensitivity analysis re-defining exposure by prescription at 60-days showed very similar results to the main analysis. Because misclassification of treatment with ACEI/ARB (or not) will become more likely over time, we censored all observations at 2 years after baseline AKI; median follow-ups were 1.1 years in England and 1.4 years in Sweden for the heart-failure-outcome analysis.

Our results show a very high rate of death in this vulnerable patient group: In England, 25% of people who survived to 30 days after discharge from an AKI admission subsequently died during a median follow-up of 1.1 years, similar to the 27% in 1.4 years’ follow-up in Sweden. It is plausible that the increased risk of death seen in those who stopped ACEI/ARB in the English cohort may reflect withdrawal of medication in the sickest people who were being managed with supportive care, which we were unable to fully account for in our multivariable models. This is supported by the hazard ratio for death decreasing towards the null when stratified by follow-up time, although this result may also represent survivor bias owing to ‘depletion of susceptibles’ [21]. Furthermore, the association of stopping ACEI/ARB and cumulative incidence of heart failure, AKI, and stroke will be affected by the high competing risk of death from other causes. However, we chose to undertake our analysis based on the cause-specific hazard which is appropriate for investigating the causal relationship between therapy use and outcomes [22, 23]. We chose not to examine death from specific causes as we anticipated that if frailty was the underlying mechanism we would see an association between stopping ACEI/ARB and both cardiovascular and non-cardiovascular causes of death.

A previous population-based cohort study also looked at re-admissions and death among a mixed population of ACEI/ARB users and non-users who experienced AKI [24]. This study described a protective association between ACEI/ARB use post-AKI discharge and death (HR 0.85, 95% CI 0.81–0.89), but contrary to our findings found a higher risk of hospitalisation for a composite outcome that included kidney events and heart failure (HR 1.28, 95% CI 1.12–1.46). However, this study included people as exposed if they received an ACEI/ARB prescription in the first 6 months making these results vulnerable to exposure misclassification and time-dependent confounding. Our results, which show extremely high rates of death and other outcomes up to 2 years after the baseline AKI admission, demonstrate the importance of outcomes in this period after AKI. Another recent study examined outcomes for people who experienced an episode of AKI (defined by creatinine changes, in contrast with our work) and were non-ACEI/ARB users before the episode, without diagnosed heart failure [25]. Consistent with our results, this study showed no increase in subsequent risk of AKI among people who were exposed to ACEI/ARB after discharge (adjusted odds ratio 0.71, 95% CI 0.45–1.12). Our work adds to these studies by looking specifically at a range of outcomes among people who were ACEI/ARB users before they experienced AKI and is therefore closely aligned with the common clinical dilemma we sought to study.

Our results support evidence that people who use ACEI/ARB and are hospitalised for AKI are at high risk of hospital admission with heart failure, stroke, or recurrent AKI, but suggest that this is not predominantly because of withdrawal of ACEI/ARB [2]. Our study only included ACEI/ARB users who experience AKI and does not relate to the rate of AKI among ACEI/ARB users, which we have previously shown to be 6.4/1000 person-years (95% CI 6.30–6.50) in England, with only a 12% higher rate among ACEI/ARB users compared to people taking other antihypertensives [26].

## Conclusion

We found no association between stopping ACEI/ARB after hospitalisation for AKI with important medium-term kidney and cardiovascular outcomes. Further research in cohorts which include data on in-hospital and discharge prescribing, linked to inpatient and outpatient kidney function data, would further increase our understanding. Clinicians can be reassured that restarting ACEI/ARB medications in people with evidence-based indications for long-term treatment after discharge with AKI is not associated with harm.

## Supplementary information

**Additional file 1 : Figure S1.** Study design diagram, English cohort. **Table S1.** Top 10 admission codes for baseline AKI admission in English cohort. **Table S2.** Baseline characteristics of English and Swedish cohorts (overall and heart failure outcome analysis). **Figure S2.** Represcribing ACEI/ARB in both cohorts. **Figure S3.** Represcribing by year in English cohort. **Table S3.** Baseline characteristics of people censored during immortal time, both cohorts. **Table S4.** Absolute rates and hazard ratios for all outcomes, both cohorts. **Table S5**. Model building for both cohorts. **Figure S4.**, **Table S6.** Propensity score analysis. **Table S7.** Main and sensitivity analyses results, heart failure outcome. **Table S8.** Main and sensitivity analyses results, acute kidney injury outcome. **Table S9.** Main and sensitivity analyses, stroke outcome. **Table S10.** Main and sensitivity analyses, mortality outcome. **Figure S5.** Summary forest plot of all main and sensitivity analyses, all outcomes.

## Data Availability

The datasets supporting the conclusions for the English cohort are not publicly available due to CPRD licencing restrictions. All codes used in the English analysis are available on the Electronic Health Records Research Group Data Compass website: https://datacompass.lshtm.ac.uk/1484/. The datasets supporting the conclusions for the Swedish cohort are not publicly available; however, study materials are available to other researchers for collaborative projects. Requests should be sent to the SCREAM steering committee group (contact: juan.jesus.carrero@ki.se).

## Abbreviations

ACEI/ARB: Angiotensin-converting enzyme inhibitors/angiotensin II receptor blockers
AKI: Acute kidney injury
BMI: Body mass index
CPRD: Clinical Practice Research Datalink
eFI: Electronic frailty index
eGFR: Estimated glomerular filtration rate
ESRD: End-stage renal disease
HES: Hospital Episode Statistics
HFrEF: Heart failure with reduced ejection fraction
HR: Hazard ratio
ICD: International Classification of Diseases
IQR: Interquartile range
NHS: National Health Service
PS: Propensity score
SCREAM: Stockholm CREAtinine Measurements
SMD: Standardised mean differences
UK: United Kingdom
