# Supplementary material for: Stopping renin-angiotensin system blockers after acute kidney injury and risk of adverse outcomes: parallel population-based cohort studies in English and Swedish routine care
Source: BMC Med. 2020 Jul 29;18:195. doi: 10.1186/s12916-020-01659-x (PMC7389346; doi:10.1186/s12916-020-01659-x)
Supplement: Supplementary file 1 — Additional file 1 : Figure S1. Study design diagram, English cohort. Table S1. Top 10 admission codes for baseline AKI admission in English cohort. Table S2. Baseline characteristics of English and Swedish cohorts (overall and heart failure outcome analysis). Figure S2. Represcribing ACEI/ARB in both cohorts. Figure S3. Represcribing by year in English cohort. Table S3. Baseline characteristics of people censored during immortal time, both cohorts. Table S4. Absolute rates and hazard ratios for all outcomes, both cohorts. Table S5. Model building for both cohorts. Figure S4., Table S6. Propensity score analysis. Table S7. Main and sensitivity analyses results, heart failure outcome. Table S8. Main and sensitivity analyses results, acute kidney injury outcome. Table S9. Main and sensitivity analyses, stroke outcome. Table S10. Main and sensitivity analyses, mortality outcome. Figure S5. Summary forest plot of all main and sensitivity analyses, all outcomes. [file 12916_2020_1659_MOESM1_ESM.docx]

**Additional file 1:**

**Stopping renin-angiotensin system blockers after AKI and risk of adverse outcomes: parallel population-based cohort studies in English and Swedish routine care**

**Table of Contents**

Figure S12

Table S13

Table S24-5

Figure S26

Figure S37

Table S38-9

Table S410

Table S511-12

Sensitivity analyses – methods13-14

Figure S415

Table S616-18

Table S719

Table S820

Table S921

Table S1022

Figure S523

**
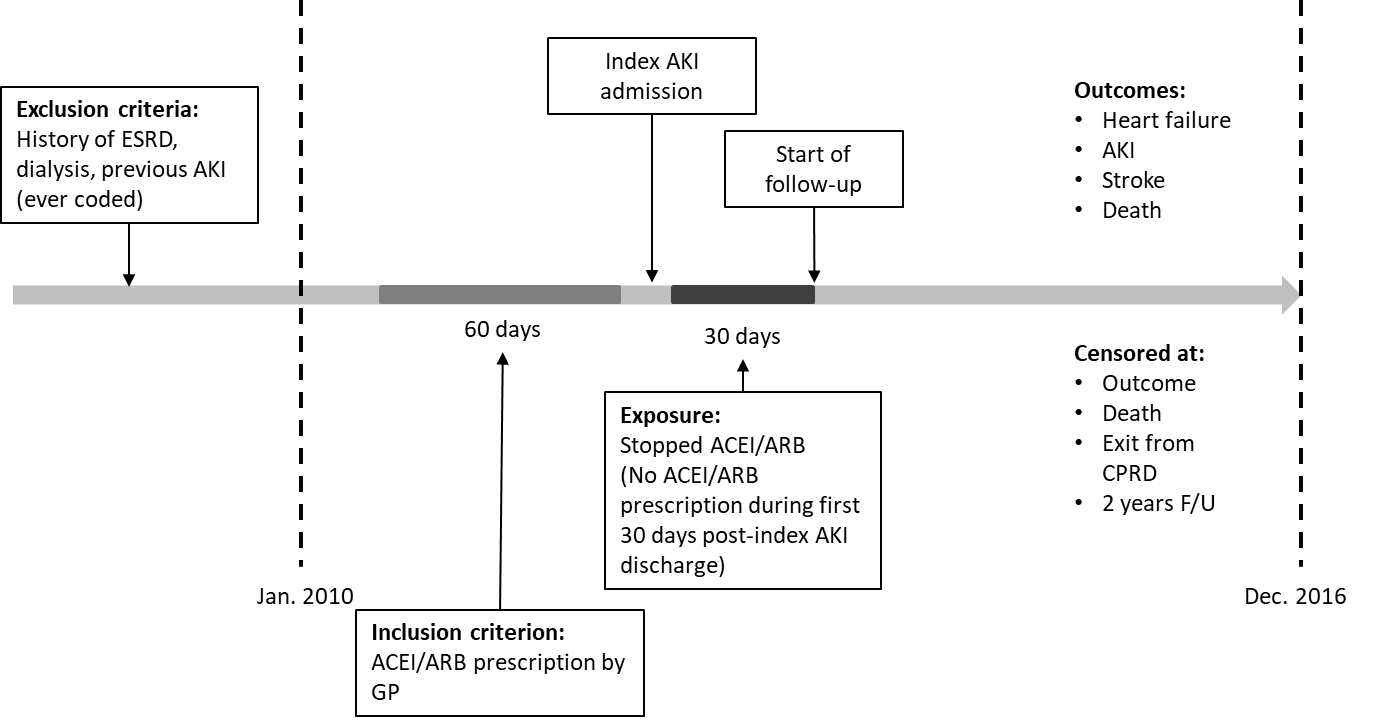
**

**Figure S1.** Diagram of study design in the English cohort

ESRD-End-stage renal disease; AKI-acute kidney injury; ACEI/ARB-ACE Inhibitors or angiotensin receptor blocker; CPRD-Clinical Practice Research Datalink; F/U-follow-up.

| **Diagnosis** | **ICD-10 code in 1^st^ diagnostic position of baseline AKI admission** | **n (%)** |
| --- | --- | --- |
| Acute renal failure | N17 | 3262 (38) |
| Other disorders of urinary system | N39 | 579 (6.8) |
| Pneumonia, organism unspecified | J18 | 421 (4.9) |
| Other gastroenteritis and colitis of infectious and unspecified origin | A09 | 260 (3.0) |
| Other sepsis | A41 | 177 (2.1) |
| Heart failure | I50 | 171 (2.0) |
| Unspecified acute lower respiratory infection | J22 | 157 (1.8) |
| Cellulitis | L03 | 148 (1.7) |
| Volume depletion | E86 | 140 (1.6) |
| Other chronic obstructive pulmonary disease | J44 | 138 (1.6) |
| Other |  | 3113 (36) |
| **Total** |  | **8566 (100)** |

**Table S1.** The ten most commonly recorded ICD-10 codes in the primary diagnostic position in HES for the baseline AKI admission for the English Cohort

|  |  | **English cohort discharged from hospital after AKI admission** | **English cohort in heart failure outcome analysis** | **Swedish cohort discharged from hospital after AKI admission** | **Swedish cohort in heart failure outcome analysis** |
| --- | --- | --- | --- | --- | --- |
|  |  | N=8566 | N=7303 | N=2024 | N=1790 |
| **Age, mean (SD)** | | 78 (11) | 77 (11) | 76 (12) | 75 (12) |
| **Age** | 18-69 | 1811 (21) | 1670 (23) | 606 (30) | 572 (32) |
|  | 70-74 | 990 (12) | 868 (12) | 250 (12) | 235 (13) |
|  | 75-79 | 1516 (18) | 1340 (18) | 280 (14) | 241 (13) |
|  | 80-84 | 1750 (20) | 1499 (21) | 366 (18) | 308 (17) |
|  | 85-89 | 1497 (18) | 1207 (17) | 339 (17) | 286 (16) |
|  | ≥90 | 1002 (12) | 719 (10) | 183 (9) | 148 (8) |
| **Women** | | 4103 (48) | 3431 (47) | 919 (45) | 805 (45) |
| **Baseline eGFR (mls/min/1.73m^2^), mean (SD)** | | 53 (22) | 54 (22) | 54 (24) | 54 (24) |
| **eGFR category** | No known CKD | 1043 (12) | 875 (12) | 139 (7) | 127 (7) |
|  | G1-No CKD | 461 (5) | 416 (6) | 152 (8) | 144 (8) |
|  | G2-Mild | 2178 (25) | 1893 (26) | 572 (28) | 521 (29) |
|  | G3a-Mild Mod | 1829 (21) | 1564 (21) | 400 (20) | 355 (20) |
|  | G3b-Mod-Severe | 1992 (23) | 1686 (23) | 414 (20) | 354 (20) |
|  | G4-Severe | 981 (12) | 801 (11) | 284 (14) | 236 (13) |
|  | G5-Kidney failure | 82 (1) | 68 (1) | 63 (3) | 53 (3) |
| **Comorbidities** | Arrhythmia | 2763 (32) | 2249 (31) | 753 (37) | 685 (38) |
|  | Diabetes | 3897 (46) | 3420 (47) | 833 (41) | 766 (43) |
|  | Heart failure | 2728 (32) | 2213 (30) | 937 (46) | 848 (47) |
|  | Hypertension | 7398 (86) | 6339 (87) | 1633 (81) | 1550 (87) |
|  | IHD | 4457 (52) | 3751 (51) | 728 (36) | 658 (37) |
| **Medications** | β blockers | 3644 (43) | 3096 (42) | 1339 (66) | 1214 (68) |
|  | CCB | 2973 (35) | 2610 (36) | 658 (33) | 657 (37) |
|  | Loop diuretics | 5053 (59) | 4215 (58) | 532 (26) | 499 (28) |
|  | Spironolactone | 1024 (12) | 848 (12) | 488 (24) | 423 (24) |
| **Year of discharge** | 2006 |  |  | 173 (9) | 153 (9) |
|  | 2007 |  |  | 199 (10) | 170 (9) |
|  | 2008 |  |  | 249 (12) | 218 (12) |
|  | 2009 |  |  | 300 (15) | 278 (16) |
|  | 2010 | 995 (12) | 864 (12) | 339 (17) | 303 (17) |
|  | 2011 | 1121 (13) | 961 (13) | 341 (17) | 315 (18) |
|  | 2012 | 1343 (16) | 1153 (16) | 423 (21) | 353 (20) |
|  | 2013 | 1436 (17) | 1213 (17) |  |  |
|  | 2014 | 1436 (17) | 1228 (17) |  |  |
|  | 2015 | 1314 (15) | 1104 (15) |  |  |
|  | 2016 | 921 (11) | 780 (11) |  |  |
| **Ethnicity** | White | 8077 (94) | 6870 (94) |  |  |
|  | Other | 411 (5) | 369 (4) |  |  |
|  | Missing | 78 (1) | 64 (1) |  |  |
| **Smoking** | Non-smoker | 2626 (31) | 2197 (30) |  |  |
|  | Ex-smoker | 4982 (58) | 4293 (59) |  |  |
|  | Current smoker | 933 (11) | 793 (11) |  |  |
|  | Missing | 25 (0.3) | 20 (0.3) |  |  |
|  |  | **English cohort discharged from hospital after AKI admission** | **English cohort in heart failure outcome analysis** | **Swedish cohort discharged from hospital after AKI admission** | **Swedish cohort in heart failure outcome analysis** |
|  |  |  |  |  |  |
| **Alcohol use** | Non-drinker | 990 (12) | 822 (11) |  |  |
|  | Ex-drinker | 1507 (18) | 1295 (18) |  |  |
|  | Current drinker | 5635 (66) | 4845 (66) |  |  |
|  | Missing | 434 (5) | 341 (5) |  |  |
| **BMI (kg/m^2^), mean (SD)** | | 29 (7) | 29 (7) |  |  |
| **BMI** | BMI <18.5 | 211 (2) | 167 (2) |  |  |
|  | BMI 18.5-24.9 | 2200 (26) | 1789 (24) |  |  |
|  | BMI 25-29.9 | 2719 (32) | 2339 (32) |  |  |
|  | BMI ≥30 | 3093 (36) | 2749 (38) |  |  |
|  | Missing | 343 (4) | 259 (4) |  |  |
| **Dialysis during baseline AKI admission** | | 309 (4) | 261 (4) |  |  |
| **eFI** | Fit | 455 (5) | 417 (6) |  |  |
|  | Mild frailty | 2440 (28) | 2136 (29) |  |  |
|  | Moderate frailty | 3812 (45) | 3228 (44) |  |  |
|  | Severe frailty | 1859 (22) | 1522 (21) |  |  |

**Table S2**. Baseline characteristics of the English and Swedish cohorts on discharge from hospital and overall cohorts included in the heart failure (primary outcome) analysis.

Data are % unless otherwise specified. SD-standard deviation; BMI – body mass index; eGFR – estimated glomerular filtration rate; IHD-ischaemic heart disease; CCB-calcium channel blockers. BMI, alcohol use, smoking and ethnicity were not available in the Swedish dataset. Characteristics stratified by treatment group from Sweden were derived at 30 days after discharge.

**A - England**


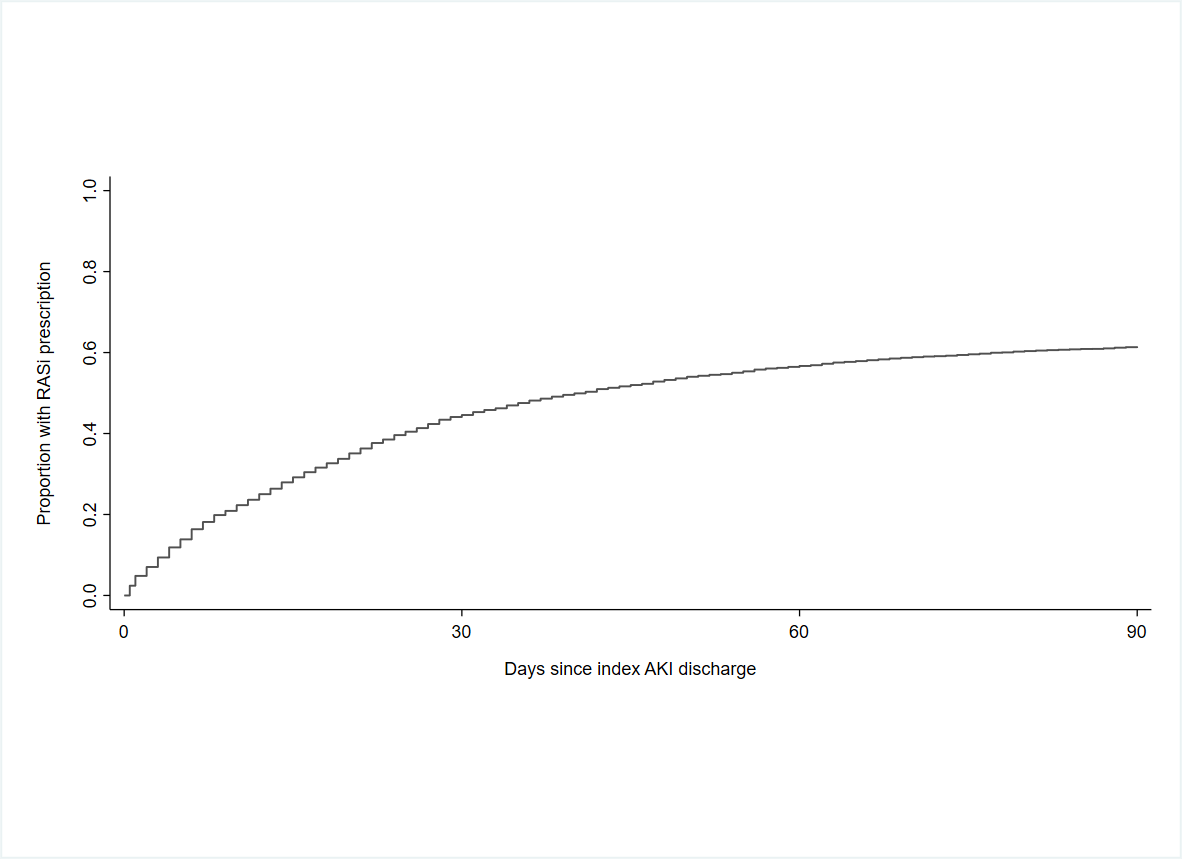


**B - Sweden**


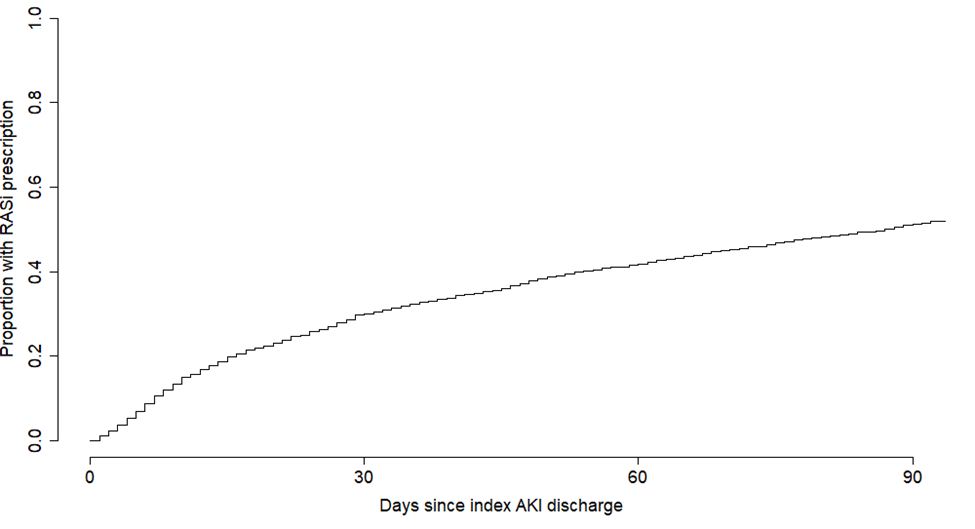


**Figure S2.** Proportion of participants prescribed ACEI/ARB following discharge from admission with AKI in England (A: top) and Sweden (B: bottom)


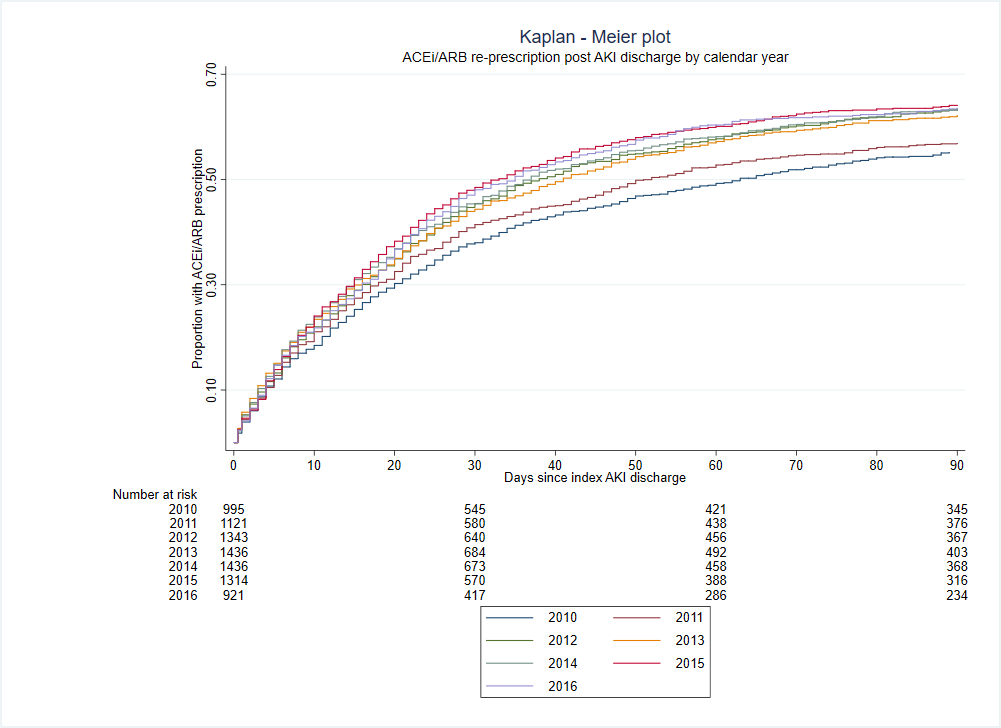


**Figure S3.** Proportion of participants prescribed ACEI/ARB in English Primary Care following discharge from admission with AKI, stratified by year

|  | **Outcome** | **Heart failure** | **AKI** | **Stroke** | **Death** |
| --- | --- | --- | --- | --- | --- |
|  |  | N=1263 | N=1383 | N=1128 | N=1100 |
| **Age (years), mean (SD)** | | 82 (10) | 81 (11) | 82 (10) | 82 (10) |
| **Age** | 18-69 | 141 (11) | 200 (15) | 127 (11) | 120 (11) |
|  | 70-74 | 122 (10) | 130 (9) | 103 (9) | 101 (9) |
|  | 75-79 | 176 (14) | 188 (14) | 153 (14) | 149 (14) |
|  | 80-84 | 251 (20) | 269 (20) | 212 (19) | 205 (19) |
|  | 85-89 | 290 (23) | 316 (23) | 265 (24) | 262 (24) |
|  | ≥90 | 283 (22) | 280 (20) | 268 (24) | 263 (24) |
| **Women** |  | 672 (53) | 734 (53) | 603 (54) | 590 (54) |
| **Baseline eGFR (mls/min/1.73m^2^), mean (SD)** | | 50 (21) | 51 (21) | 51 (21) | 51 (21) |
| **eGFR category** | No known CKD | 168 (13) | 188 (14) | 157 (14) | 154 (14) |
|  | G1-No CKD | 45 (4) | 57 (4) | 43 (4) | 42 (4) |
|  | G2-Mild | 285 (23) | 309 (22) | 262 (23) | 254 (23) |
|  | G3a-Mild-Mod | 265 (21) | 306 (22) | 251 (22) | 242 (22) |
|  | G3b-Mod-Severe | 306 (24) | 318 (23) | 250 (22) | 244 (22) |
|  | G4-Severe | 180 (14) | 190 (14) | 152 (14) | 151 (14) |
|  | G5-Kidney failure | 14 (1) | 15 (1) | 13 (1) | 13 (1) |
| **Comorbidities** | Arrhythmia | 514 (41) | 508 (37) | 424 (38) | 409 (37) |
|  | Diabetes | 477 (38) | 541 (39) | 427 (38) | 411 (37) |
|  | Heart failure | 515 (41) | 486 (35) | 403 (36) | 393 (36) |
|  | Hypertension | 1059 (84) | 1167 (84) | 952 (84) | 931 (85) |
|  | IHD | 706 (56) | 711 (51) | 593 (53) | 579 (53) |
| **Medications** | Beta blockers | 548 (43) | 571 (41) | 453 (40) | 442 (40) |
|  | CCB | 363 (29) | 428 (31) | 338 (30) | 328 (30) |
|  | Loop diuretics | 838 (66) | 864 (63) | 723 (64) | 706 (64) |
|  | Spironolactone | 176 (14) | 153 (11) | 122 (11) | 118 (11) |
| **Ethnicity** | White | 1207 (96) | 1320 (95) | 1081 (96) | 1054 (96) |
|  | Other | 42 (3) | 47 (3) | 33 (3) | 32 (3) |
|  | Missing | 14 (1) | 16 (1) | 14 (1) | 14 (1) |
| **Smoking** | Non-smoker | 429 (34) | 445 (32) | 383 (34) | 373 (34) |
|  | Ex-smoker | 689 (55) | 773 (56) | 610 (54) | 596 (54) |
|  | Current smoker | 140 (11) | 160 (12) | 131 (12) | 127 (12) |
|  | Missing | 5 (<1) | 5 (<1) | 4 (<1) | 4 (<1) |
| **Alcohol use** | Non-drinker | 168 (13) | 177 (13) | 151 (13) | 148 (13) |
|  | Ex-drinker | 212 (17) | 235 (17) | 182 (16) | 175 (16) |
|  | Current drinker | 790 (63) | 880 (64) | 715 (63) | 698 (63) |
|  | Missing | 93 (7) | 91 (7) | 80 (7) | 79 (7) |
| **BMI (kg/m^2^), mean (SD)** | | 28 (7) | 28 (7) | 28 (7) | 28 (7) |
| **BMI** | BMI <18.5 | 44 (3) | 49 (4) | 41 (4) | 41 (4) |
|  | BMI 18.5-24.9 | 411 (33) | 434 (31) | 368 (33) | 361 (33) |
|  | BMI 25-29.9 | 380 (30) | 413 (30) | 337 (30) | 324 (29) |
|  | BMI ≥30 | 344 (27) | 399 (29) | 299 (27) | 293 (27) |
|  | Missing | 84 (7) | 88 (6) | 83 (7) | 81 (7) |
| **Dialysis during baseline AKI admission** | | 48 (4) | 56 (4) | 42 (4) | 41 (4) |

**Table S3A.** Baseline characteristics of people in the English cohorts for each outcome censored during the first 30 days after discharge following admission with AKI

Data are % unless otherwise specified. SD-standard deviation; BMI – body mass index; eGFR – estimated glomerular filtration rate; IHD-ischaemic heart disease; CCB-calcium channel blockers.

|  | **Outcome** | **Heart failure** | **AKI** | **Stroke** | **Death** |
| --- | --- | --- | --- | --- | --- |
|  |  | N=234 | N=180 | N=130 | N=121 |
| **Age (years), mean (SD)** | | 80 (9) | 78 (11) | 80 (9) | 80 (9) |
| **Age** | 18-69 | 34 (15) | 36 (20) | 15 (12) | 15 (12) |
|  | 70-74 | 15 (6) | 15 (8) | 11 (8) | 8 (7) |
|  | 75-79 | 39 (17) | 32 (18) | 22 (17) | 22 (18) |
|  | 80-84 | 58 (25) | 43 (24) | 35 (27) | 34 (28) |
|  | 85-89 | 53 (23) | 34 (19) | 27 (21) | 25 (21) |
|  | ≥90 | 35 (15) | 20 (11) | 20 (15) | 17 (14) |
| **Women** |  | 114 (49) | 76 (42) | 67 (52) | 62 (51) |
| **Baseline eGFR (mls/min/1.73m^2^), mean (SD)** | | 47 (22) | 51 (21) | 51 (24) | 51 (25) |
| **eGFR category** | No known CKD | 12 (5) | 10 (6) | 4 (3) | 3 (2) |
|  | G1-No CKD | 8 (3) | 11 (6) | 6 (5) | 6 (5) |
|  | G2-Mild | 51 (22) | 46 (26) | 39 (30) | 36 (30) |
|  | G3a-Mild-Mod | 45 (19) | 31 (17) | 24 (18) | 22 (18) |
|  | G3b-Mod-Severe | 60 (26) | 41 (23) | 24 (18) | 23 (19) |
|  | G4-Severe | 48 (21) | 30 (17) | 25 (19) | 23 (19) |
|  | G5-Kidney failure | 10 (4) | 11 (6) | 8 (6) | 8 (7) |
| **Comorbidities** | Arrhythmia | 80 (34) | 24 (13) | 6 (5) | 0 (0) |
|  | Diabetes | 69 (29) | 55 (31) | 33 (25) | 29 (24) |
|  | Heart failure | 113 (48) | 32 (18) | 5 (4) | 0 (0) |
|  | Hypertension | 94 (40) | 53 (29) | 9 (7) | 0 (0) |
|  | IHD | 75 (32) | 24 (13) | 6 (5) | 0 (0) |
| **Medications** | Beta blockers | 174 (74) | 124 (69) | 87 (67) | 80 (66) |
|  | CCB | 71 (30) | 60 (33) | 38 (29) | 35 (29) |
|  | Loop diuretics | 94 (40) | 61 (34) | 43 (33) | 42 (35) |
|  | Spironolactone | 95 (41) | 47 (26) | 34 (26) | 31 (26) |

**Table S3B.** Baseline characteristics of people in the Swedish cohorts for each outcome censored during the first 30 days after discharge following admission with AKI

Data are % unless otherwise specified. SD-standard deviation; eGFR – estimated glomerular filtration rate; IHD-ischaemic heart disease; CCB-calcium channel blockers.

| **Outcome** | **Exposure** | **Events** | **Total follow-up (per 1000 person years)** | **Crude Rate**  **(per 1000 person years)**  **(95% CI)** | **Crude**  **Hazard Ratio**  **(95% CI)** | **Adjusted Hazard Ratio**  **(95% CI)** |
| --- | --- | --- | --- | --- | --- | --- |
| **Swedish analyses** | | |  |  |  |  |
| Heart Failure | Continued  ACEI/ARB | 126 | 0.63 | 201.3  (167.7-239.6) | 1 | 1 |
|  | Stopped  ACEI/ARB | 219 | 1.46 | 150.5  (131.3-171.8) | 0.76  (0.61-0.95) | 0.91  (0.73-1.13) |
| Acute Kidney Injury | Continued  ACEI/ARB | 38 | 0.71 | 53.3  (37.8-73.2) | 1 | 1 |
|  | Stopped  ACEI/ARB | 64 | 1.58 | 40.4  (31.1-51.6) | 0.76  (0.51-1.14) | 0.81  (0.54-1.21) |
| Stroke | Continued  ACEI/ARB | 29 | 0.74 | 39.0  (26.1-56.0) | 1 | 1 |
|  | Stopped  ACEI/ARB | 35 | 1.65 | 21.3  (14.8-29.6) | 0.55  (0.33-0.89) | 0.56  (0.34-0.93) |
| Death | Continued  ACEI/ARB | 174 | 0.77 | 227.3  (194.8-263.7) | 1 | 1 |
|  | Stopped  ACEI/ARB | 342 | 1.68 | 203.6  (182.6-226.3) | 0.90  (0.75-1.08) | 0.94  (0.78-1.13) |
| **English analyses** | | |  |  |  |  |
| Heart Failure | Continued  ACEI/ARB | 287 | 3.76 | 76.3  (67.9-85.6) | 1 | 1 |
|  | Stopped  ACEI/ARB | 324 | 4.45 | 72.8  (65.3-81.1) | 0.96  (0.82-1.12) | 1.10  (0.93-1.30) |
| Acute Kidney Injury | Continued  ACEI/ARB | 349 | 3.70 | 94.3  (84.9-104.8) | 1 | 1 |
|  | Stopped  ACEI/ARB | 367 | 4.33 | 84.7  (76.5-93.9) | 0.90  (0.78-1.04) | 0.90  (0.77-1.05) |
| Stroke | Continued  ACEI/ARB | 73 | 4.00 | 18.3  (14.5-23.0) | 1 | 1 |
|  | Stopped  ACEI/ARB | 91 | 4.69 | 19.4  (15.8-23.8) | 1.06  (0.78-1.45) | 0.99  (0.71-1.38) |
| Death | Continued  ACEI/ARB | 767 | 4.04 | 189.9  (176.9-203.8) | 1 | 1 |
|  | Stopped  ACEI/ARB | 1118 | 4.75 | 235.3  (221.9-249.5) | 1.24  (1.13-1.36) | 1.27  (1.15-1.41) |

**Table S4.** Numbers of events, absolute rates and hazard ratios for all outcomes comparing people who stop or continue ACEI/ARB after an admission with AKI

| **Variable included in Cox model** |  | **Age and sex adjusted** | **Age, sex, eGFR**  **and comorbidity adjusted** | **Fully adjusted** |
| --- | --- | --- | --- | --- |
|  |  | N=7303 | N=7303 | N=6747 |
| **Exposure** | Continued ACEI/ARB | Reference | Reference | Reference |
|  | Stopped ACEI/ARB | 0.96 (0.82-1.13) | 1.07 (0.91-1.26) | 1.10 (0.93-1.30) |
| **Age (per year increase)** | | 1.03 (1.02-1.04) | 1.01 (1.00-1.02) | 1.01 (1.00-1.03) |
| **Sex** | Male | Reference | Reference | Reference |
|  | Female | 0.89 (0.75-1.04) | 0.98 (0.83-1.15) | 0.91 (0.76-1.09) |
| **eGFR category** | G1-No CKD |  | Reference | Reference |
|  | G2-Mild |  | 1.20 (0.69-2.09) | 1.05 (0.59-1.87) |
|  | G3a-Mild Mod |  | 1.47 (0.85-2.56) | 1.34 (0.76-2.39) |
|  | G3b-Mod-Severe |  | 1.74 (1.00-3.01) | 1.47 (0.83-2.60) |
|  | G4-Severe |  | 2.08 (1.18-3.66) | 1.70 (0.94-3.07) |
|  | G5-Kidney failure |  | 2.65 (1.06-6.57) | 2.14 (0.81-5.68) |
|  | No known CKD |  | 0.97 (0.53-1.79) | 0.77 (0.40-1.49) |
| **Comorbidities** | Arrhythmia |  | 1.76 (1.48-2.10) | 1.85 (1.54-2.22) |
|  | Diabetes |  | 1.08 (0.92-1.27) | 1.05 (0.88-1.25) |
|  | Heart failure |  | 4.30 (3.54-5.21) | 3.67 (2.99-4.52) |
|  | Hypertension |  | 1.32 (1.04-1.68) | 1.20 (0.93-1.56) |
|  | IHD |  | 1.36 (1.13-1.63) | 1.37 (1.12-1.67) |
| **Medications** | Beta blockers |  |  | 1.14 (0.96-1.36) |
|  | CCB |  |  | 1.09 (0.91-1.30) |
|  | Loop diuretics |  |  | 1.81 (1.44-2.27) |
| **Ethnicity** | White |  |  | 0.71 (0.50-1.02) |
| **Smoking status** | Non-smoker |  |  | Reference |
|  | Current smoker |  |  | 1.20 (0.86-1.67) |
|  | Ex-smoker |  |  | 1.06 (0.87-1.30) |
| **Alcohol use** | Non-drinker |  |  | Reference |
|  | Current drinker |  |  | 0.78 (0.61-1.01) |
|  | Ex-drinker |  |  | 0.87 (0.65-1.16) |
| **BMI (per kg/m^2^ increase)** | |  |  | 1.01 (1.00-1.02) |
| **Year of discharge** | 2010 |  |  | Reference |
|  | 2011 |  |  | 1.05 (0.77-1.42) |
|  | 2012 |  |  | 0.91 (0.67-1.23) |
|  | 2013 |  |  | 0.91 (0.67-1.23) |
|  | 2014 |  |  | 1.18 (0.88-1.59) |
|  | 2015 |  |  | 1.05 (0.76-1.45) |
|  | 2016 |  |  | 0.79 (0.52-1.20) |

**Table S5A.** Full model including HR for all included covariates for admission with Heart Failure (primary outcome) for the English cohort comparing those who stop ACEI/ARB after AKI discharge, with those who continue

BMI – body mass index; eGFR – estimated glomerular filtration rate; IHD-ischaemic heart disease; CCB-calcium channel blockers.

| **Variable included in Cox model** |  | **Age and sex adjusted** | **Age, sex, eGFR and comorbidity adjusted** | **Fully adjusted** |
| --- | --- | --- | --- | --- |
|  |  | N=1790 | N=1790 | N=1790 |
| **Exposure** | Continued ACEI/ARB | Reference | Reference | Reference |
|  | Stopped ACEI/ARB | 0.75 (0.60-0.94) | 0.88 (0.71-1.10) | 0.91 (0.73-1.13) |
| **Age (per year)** | | 1.04 (1.03-1.05) | 1.01 (1.00-1.02) | 1.01 (1.00-1.02) |
| **Sex** | Male | Reference | Reference | Reference |
|  | Female | 1.04 (0.84-1.29) | 1.03 (0.83-1.28) | 0.98 (0.78-1.22) |
| **eGFR category** | G1-No CKD |  | Reference | Reference |
|  | G2-Mild |  | 0.79 (0.42-1.48) | 0.76 (0.40-1.43) |
|  | G3a-Mild Mod |  | 1.14 (0.60-2.15) | 1.05 (0.56-1.99) |
|  | G3b-Mod-Severe |  | 0.97 (0.52-1.83) | 0.84 (0.44-1.60) |
|  | G4-Severe |  | 1.35 (0.71-2.56) | 1.15 (0.60-2.20) |
|  | G5-Kidney failure |  | 1.02 (0.45-2.31) | 0.84 (0.36-1.93) |
|  | No known CKD |  | 1.15 (0.58-2.31) | 1.22 (0.59-2.51) |
| **Comorbidities** | Arrhythmia |  | 1.76 (1.39-2.24) | 1.55 (1.21-1.98) |
|  | Diabetes |  | 0.99 (0.79-1.24) | 0.98 (0.78-1.23) |
|  | Heart failure |  | 9.42 (6.36-13.96) | 7.20 (4.79-10.81) |
|  | Hypertension |  | 0.97 (0.74-1.27) | 0.95 (0.72-1.25) |
|  | IHD |  | 1.43 (1.14-1.79) | 1.42 (1.13-1.78) |
| **Medications** | Beta blockers |  |  | 1.32 (0.98-1.77) |
|  | CCB |  |  | 1.13 (0.88-1.46) |
|  | Loop diuretics |  |  | 1.85 (1.17-2.92) |
| **Year of discharge** | 2006 |  |  | Reference |
|  | 2007 |  |  | 1.11 (0.70-1.75) |
|  | 2008 |  |  | 1.11 (0.70-1.75) |
|  | 2009 |  |  | 1.01 (0.64-1.60) |
|  | 2010 |  |  | 1.06 (0.67-1.66) |
|  | 2011 |  |  | 1.00 (0.62-1.60) |
|  | 2012 |  |  | 1.25 (0.75-2.09) |

**Table S5B.** Full model including HR for all included covariates for admission with Heart Failure (primary outcome) for the Swedish cohort comparing those who stop ACEI/ARB after AKI discharge, with those who continue

eGFR – estimated glomerular filtration rate; IHD-ischaemic heart disease; CCB-calcium channel blockers.

### **Sensitivity analyses - Methods**

***Propensity-score matched analyses (Figures S4-5, Tables S6-10)***

To maximise comparability between our exposure groups, we conducted propensity-score matched analyses to estimate the HRs for each study outcome. We used logistic regression to calculate the odds of stopping or being prescribed ACEI/ARB by the start of follow-up for each year, adjusting for relevant covariates. Factors included in the propensity score model were all covariates described in the main text, plus in the English cohort we also adjusted for frailty using the electronic frailty index (eFI) as a covariate, including both the frailty category (fit, mildly, moderately or severely frail) and the individual components comprising the score. We did not adjust for eFI in the primary analysis to maintain comparability with the Swedish cohort where these data were not available. Participants who stopped ACEI/ARB were matched 1:1 on propensity score to participants who continued therapy, using nearest neighbour matching with a caliper of 0.2. We measured standardised mean differences (SMD) for each covariate to check for balance between groups, with an SMD <10% indicating acceptable balance. We estimated HR using Cox regression for admission with heart failure, AKI, and stroke, and for death comparing those who stopped ACEI/ARB with those who continued the drugs.

***Prescribing ACEI/ARB defined at 60 days after AKI discharge (Figure S5, Tables S7-10)***

We defined people as having stopped ACEI/ARB if they had not received an ACEI/ARB prescription in primary care by 60 days after discharge following their AKI admission (rather than 30 days in the main analysis).

***Cohort defined by 2 ACEI/ARB prescriptions in 60 days prior to AKI admission, 1 within 30 days (Figure S5, Tables S7-10)***

To increase certainty that participants were regular users of ACEI/ARB, we restricted the cohort to people who had at least two prescriptions for ACEI/ARB within 60 days before the baseline AKI admission, one of which was within 30 days before admission (England only).

***Outcome events defined from first diagnostic position only (Figure S5, Tables S7-10)***

We restricted outcome events to those recorded in the first diagnostic position in HES or Swedish hospital records only (in the main analysis we used the first and second diagnostic position).

***Excluding people who required dialysis during admission (Figure S5, Tables S7-10)***

We determined HRs for all outcomes after excluding people who required dialysis during their baseline AKI admission (England only).

***Stratified follow-up time intervals (Figure S5, Tables S7-10)***

We conducted stratified analysis in three time intervals (0-29, 30-89 and 90+ days after start of follow-up) to consider changes over time in the risk of re-admission and death in participants who stopped ACEI/ARB compared with those who continued.

***Excluding people who were readmitted in the 30 days after discharge from baseline AKI admission (Figure S5, Tables S7-10)***

We repeated all analyses excluding people who were readmitted to hospital in the first 30 days after the index AKI discharge.

***
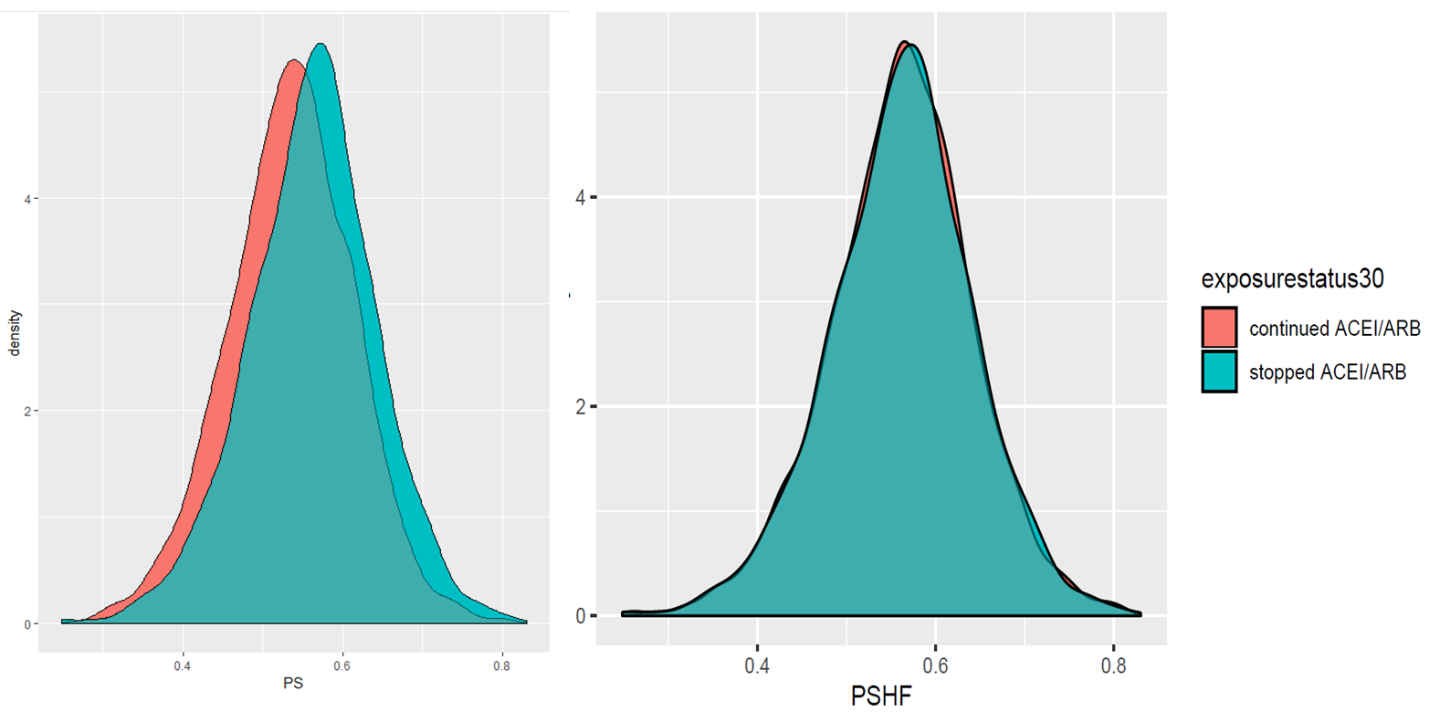
***


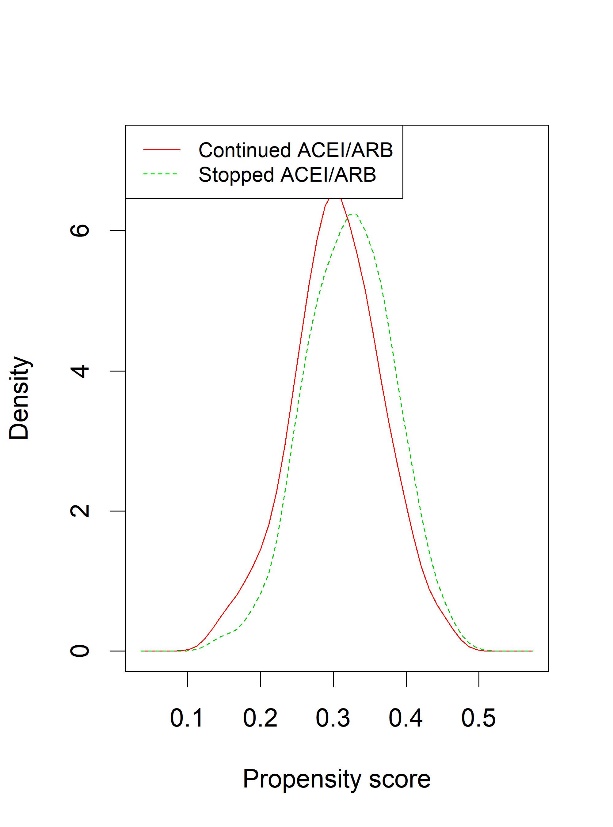

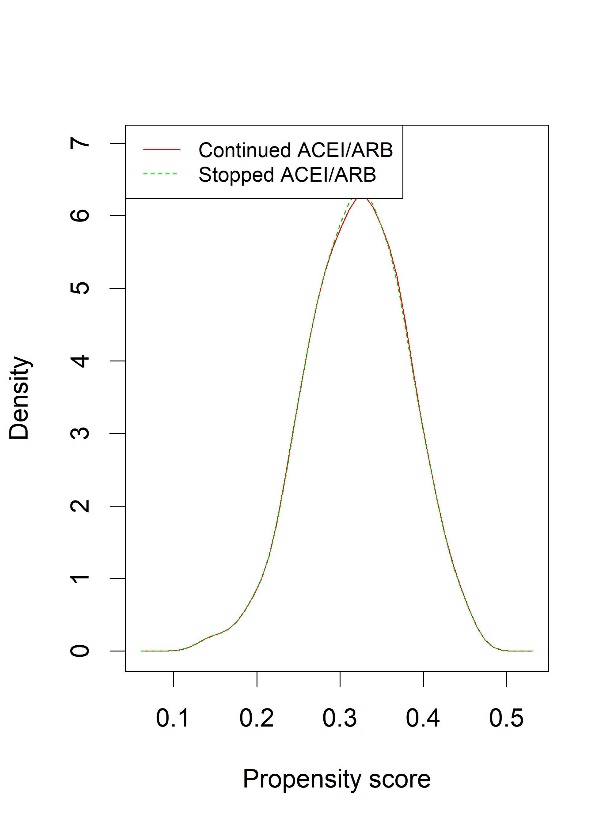


**Figure S4.** Propensity score (PS) distribution between exposure groups before (left) and after (right) PS matching in the English cohort (top figures) and Swedish cohort (bottom figures)

|  |  | **Continued ACEI/ARB** | **Stopped ACEI/ARB** |  |  |
| --- | --- | --- | --- | --- | --- |
|  |  | **n=3741** | **n=3741** | **SMD before PS** | **SMD after PS matching** |
| **Age (years), mean (SD)** | | 76.8 (11) | 76.6 (11) | 0.5 | <0.1 |
| **Female** |  | 1569 (48) | 1862 (47) | 2.1 | <0.1 |
| **eGFR category** |  |  |  | 14.6 | <0.1 |
|  | No known CKD | 476 (13) | 442 (12) |  |  |
|  | G1-No CKD | 186 (5) | 201 (5) |  |  |
|  | G2-Mild | 928 (25) | 936 (25) |  |  |
|  | G3a-Mild Moderate | 740 (20) | 792 (21) |  |  |
|  | G3b-Moderate Severe | 884 (24) | 856 (23) |  |  |
|  | G4-Severe | 489 (13) | 470 (13) |  |  |
|  | G5-Kidney failure | 38 (1) | 44 (1) |  |  |
| **Comorbidities** | Arrhythmia | 1149 (31) | 1099 (29) | 6.3 | <0.1 |
|  | Diabetes | 1752 (47) | 1746 (47) | 5.6 | <0.1 |
|  | Heart failure | 1112 (30) | 1076 (29) | 8.1 | <0.1 |
|  | Hypertension | 3271 (87) | 3271 (87) | 0.8 | <0.1 |
|  | Ischaemic heart disease | 1854 (50) | 1889 (51) | 8.3 | <0.1 |
| **Medications** | Beta blockers | 1562 (42) | 1548 (41) | 6.0 | <0.1 |
|  | Calcium channel blockers | 1393 (37) | 1357 (36) | 3.2 | <0.1 |
|  | Diuretics (excluding spironolactone) | 2127 (57) | 2123 (57) | 4.2 | <0.1 |
| **Ethnicity** | White | 3058 (93) | 3812 (95) | 10.8 | <0.1 |
| **Smoking** |  |  |  | 2.8 | <0.1 |
|  | Non-smoker | 1152 (31) | 1104 (30) |  |  |
|  | Ex-smoker | 2175 (58) | 2229 (60) |  |  |
|  | Current smoker | 414 (11) | 408 (11) |  |  |
| **Alcohol use** |  |  |  | 4.5 | <0.1 |
|  | Non-drinker | 404 (10.8) | 413 (11) |  |  |
|  | Ex-drinker | 710 (19.0) | 704 (19) |  |  |
|  | Current drinker | 2627 (70.2) | 2624 (70) |  |  |
| **BMI (kg/m^2^), mean (SD)** | | 29.2 (6.8) | 29.2 (7) | 1.2 | <0.1 |
| **Year of discharge** | |  |  | 12.8 | <0.1 |
|  | 2010 | 506 (14) | 494 (13) |  |  |
|  | 2011 | 511 (14) | 515 (14) |  |  |
|  | 2012 | 555 (15) | 582 (16) |  |  |
|  | 2013 | 644 (17) | 628 (17) |  |  |
|  | 2014 | 651 (17) | 616 (17) |  |  |
|  | 2015 | 497 (13) | 524 (14) |  |  |
|  | 2016 | 377 (10) | 382 (10) |  |  |
| **eFI** |  |  |  | 9.5 | <0.1 |
|  | Fit | 242 (7) | 221 (6) |  |  |
|  | Mild frailty | 1081 (29) | 1110 (30) |  |  |
|  | Moderate frailty | 1636 (44) | 1672 (45) |  |  |
|  | Severe frailty | 782 (21) | 738 (20) |  |  |
| **eFI components** | Activity limitation | 119 (3) | 108 (3) | 2.4 | <0.1 |
|  | Anaemia and haematinic deficiency | 2113 (57) | 2078 (56) | 5.2 | <0.1 |
|  | Arthritis | 1699 (45) | 1685 (45) | 4.4 | <0.1 |
|  | Atrial fibrillation | 838 (22) | 820 (22) | 3.9 | <0.1 |
|  | Cerebrovascular disease | 687 (18) | 686 (18) | 6.5 | <0.1 |
|  | Chronic kidney disease | 2812 (75) | 2830 (76) | 8.1 | <0.1 |
|  | Dizziness | 1281 (34) | 1292 (35) | 2.0 | <0.1 |
|  | Dyspnoea | 1636 (44) | 1600 (43) | 6.6 | <0.1 |
|  | Falls | 1056 (28) | 1015 (27) | 5.0 | <0.1 |
|  | Foot problems | 532 (14) | 541 (15) | 6.2 | <0.1 |
|  | Fragility fracture | 379 (10) | 392 (11) | 2.5 | <0.1 |
|  | Hearing impairment | 1015 (27) | 968 (26) | 2.7 | <0.1 |
|  | Heart valve disease | 155 (4) | 166 (4) | 0.4 | <0.1 |
|  | Housebound | 1942 (52) | 1899 (51) | 6.4 | <0.1 |
|  | Hypotension/syncope | 781 (21) | 771 (21) | 1.2 | <0.1 |
|  | Memory and cognitive problems | 430 (12) | 438 (12) | 12.6 | <0.1 |
|  | Mobility and transfer problems | 410 (11) | 428 (11) | 0.1 | <0.1 |
|  | Palliative care | 121 (3) | 126 (3) | 1.1 | <0.1 |
|  | Parkinsonism and tremor | 99 (3) | 105 (3) | 5.8 | <0.1 |
|  | Peptic ulcer | 282 (8) | 300 (8) | 2.3 | <0.1 |
|  | Peripheral vascular disease | 494 (13) | 479 (13) | 2.6 | <0.1 |
|  | Polypharmacy | 3622 (97) | 3622 (97) | 6.1 | <0.1 |
|  | Requirement for care | 152 (4) | 159 (4) | 7.6 | <0.1 |
|  | Respiratory disease | 1451 (39) | 1407 (38) | 4.7 | <0.1 |
|  | Skin ulcer | 718 (19) | 736 (20) | 2.9 | <0.1 |
|  | Sleep disturbance | 684 (18) | 641 (17) | 1.0 | <0.1 |
|  | Social vulnerability | 309 (8) | 302 (8) | 2.7 | <0.1 |
|  | Thyroid disease | 684 (18) | 678 (18) | 4.1 | <0.1 |
|  | Urinary incontinence | 405 (11) | 416 (11) | 4.1 | <0.1 |
|  | Urinary system disease | 1943 (52) | 1915 (51) | 5.1 | <0.1 |
|  | Visual impairment | 1541 (41) | 1470 (39) | 1.3 | <0.1 |
|  | Weight loss and anorexia | 408 (11) | 385 (10) | 2.1 | <0.1 |

**Table S6A.** Baseline characteristics of exposure groups for the heart failure outcome analysis after propensity score (PS) matching, and standardised mean differences (SMD) before and after PS matching in the English cohort. 262 participants from the main cohort were not matched.

|  |  | **Continued ACEI/ARB** | **Stopped ACEI/ARB** |  |  |
| --- | --- | --- | --- | --- | --- |
|  |  | **n=547** | **n=547** | **SMD before PS** | **SMD after PS matching** |
| **Age (years), mean (SD)** | | 74.83 (12.1) | 74.55 (11.7) | <0.1 | <0.1 |
| **Female** |  | 260 (48) | 258 (47) | <0.1 | <0.1 |
| **eGFR category** |  |  |  | 18.4 | <0.1 |
|  | No known CKD | 25 (5) | 24 (4) |  |  |
|  | G1-No CKD | 38 (7) | 47 (9) |  |  |
|  | G2-Mild | 162 (30) | 160 (29) |  |  |
|  | G3a-Mild Moderate | 116 (21) | 115 (21) |  |  |
|  | G3b-Moderate Severe | 112 (21) | 115 (21) |  |  |
|  | G4-Severe | 79 (14) | 71 (13) |  |  |
|  | G5-Kidney failure | 15 (3) | 15 (3) |  |  |
| **Comorbidities** | Arrhythmia | 225 (41) | 220 (40) | 11.0 | <0.1 |
|  | Diabetes | 251 (46) | 260 (48) | 11.3 | <0.1 |
|  | Heart failure | 278 (51) | 267 (49) | 11.6 | <0.1 |
|  | Hypertension | 467 (85) | 464 (85) | <0.01 | <0.1 |
|  | Ischaemic heart disease | 210 (38) | 193 (35) | <0.01 | <0.1 |
| **Medications** | Beta blockers | 384 (70) | 371 (68) | <0.01 | <0.1 |
|  | Calcium channel blockers | 203 (37) | 202 (37) | <0.01 | <0.1 |
|  | Diuretics (excluding spironolactone) | 409 (75) | 409 (75) | 12.7 | <0.1 |
| **Year of discharge** | |  |  | 11.4 | <0.1 |
|  | 2006 | 45 (8) | 46 (8) |  |  |
|  | 2007 | 48 (9) | 37 (7) |  |  |
|  | 2008 | 67 (12) | 62 (11) |  |  |
|  | 2009 | 94 (17) | 95 (17) |  |  |
|  | 2010 | 96 (18) | 103 (19) |  |  |
|  | 2011 | 97 (18) | 104 (19) |  |  |
|  | 2012 | 100 (18) | 100 (18) |  |  |

**Table S6B.** Baseline characteristics of exposure groups for the heart failure outcome analysis after propensity score (PS) matching, and standardised mean differences (SMD) before and after PS matching in the Swedish cohort. 8 participants from the main cohort were not matched.

|  | **Number included** | **Exposure** | **Adjusted hazard ratio**  **for heart failure**  **(95% CI)** |
| --- | --- | --- | --- |
| **Swedish analyses** | | | |
| **Main Analysis** | 1790 | Continued ACEI/ARB | 1 |
|  |  | Stopped ACEI/ARB | 0.91 (0.73-1.13) |
| **Propensity score matched cohorts** | 1094 | Continued ACEI/ARB | 1 |
|  |  | Stopped ACEI/ARB | 0.86 (0.66-1.12) |
| **Prescribing ACEI/ARB defined at 60 days after AKI discharge** | 1649 | Continued ACEI/ARB | 1 |
|  |  | Stopped ACEI/ARB | 1.03 (0.81-1.31) |
| **Cohort defined by 2 ACEI/ARB prescriptions in 60 days prior to AKI admission, 1 within 30 days** | - | Continued ACEI/ARB | N/A (not available) |
|  |  | Stopped ACEI/ARB |  |
| **Outcome events defined from first diagnostic position only** | 1790 | Continued ACEI/ARB | 1 |
|  |  | Stopped ACEI/ARB | 0.92 (0.72-1.19) |
| **Excluding people who required dialysis during admission** | - | Continued ACEI/ARB | N/A (not available) |
|  |  | Stopped ACEI/ARB |  |
| **Stratified time intervals: 0-29 days after start of follow up** | 1790 | Continued ACEI/ARB | 1 |
|  |  | Stopped ACEI/ARB | 0.71 (0.43-1.20) |
| **Stratified time intervals: 30-89 days after start of follow up** |  | Continued ACEI/ARB | 1 |
|  |  | Stopped ACEI/ARB | 0.83 (0.52-1.31) |
| **Stratified time intervals: ≥90 days after start of follow up** |  | Continued ACEI/ARB | 1 |
|  |  | Stopped ACEI/ARB | 1.01 (0.76-1.35) |
| **Excluding people who were readmitted in the 30 days after discharge from baseline AKI admission** | 1445 | Continued ACEI/ARB | 1 |
|  |  | Stopped ACEI/ARB | 0.90 (0.70-1.15) |
| **English analyses** | | | |
| **Main Analysis** | 6747 | Continued ACEI/ARB | 1 |
|  |  | Stopped ACEI/ARB | 1.10 (0.93-1.30) |
| **Propensity score matched cohorts** | 5813 | Continued ACEI/ARB | 1 |
|  |  | Stopped ACEI/ARB | 1.05 (0.87-1.27) |
| **Prescribing ACEI/ARB defined at 60 days after AKI discharge** | 6252 | Continued ACEI/ARB | 1 |
|  |  | Stopped ACEI/ARB | 1.11 (0.92-1.34) |
| **Cohort defined by 2 ACEI/ARB prescriptions in 60 days prior to AKI admission, 1 within 30 days** | 4139 | Continued ACEI/ARB | 1 |
|  |  | Stopped ACEI/ARB | 1.18 (0.96-1.45) |
| **Outcome events defined from first diagnostic position only in HES** | 6747 | Continued ACEI/ARB | 1 |
|  |  | Stopped ACEI/ARB | 1.18 (0.95-1.48) |
| **Excluding people who required dialysis during admission** | 6501 | Continued ACEI/ARB | 1 |
|  |  | Stopped ACEI/ARB | 1.11 (0.94-1.32) |
| **Stratified time intervals: 0-29 days after start of follow up** | 6747 | Continued ACEI/ARB | 1 |
|  |  | Stopped ACEI/ARB | 1.32 (0.87-2.01) |
| **Stratified time intervals: 30-89 days after start of follow up** |  | Continued ACEI/ARB | 1 |
|  |  | Stopped ACEI/ARB | 1.04 (0.71-1.51) |
| **Stratified time intervals: ≥90 days after start of follow up** |  | Continued ACEI/ARB | 1 |
|  |  | Stopped ACEI/ARB | 1.05 (0.85-1.30) |
| **Excluding people who were readmitted in the 30 days after discharge from baseline AKI admission** | 5127 | Continued ACEI/ARB | 1 |
|  |  | Stopped ACEI/ARB | 1.09 (0.90-1.32) |

**Table S7.** Summary of main results in English and Swedish cohorts and all sensitivity analyses for heart failure as an outcome comparing people who stopped or continued ACEI/ARB after admission with AKI

|  | **Number included** | **Exposure** | **Adjusted hazard ratio**  **for AKI**  **(95% CI)** |
| --- | --- | --- | --- |
| **Swedish analyses** | | | |
| **Main Analysis** | 1844 | Continued ACEI/ARB | 1 |
|  |  | Stopped ACEI/ARB | 0.81 (0.54-1.21) |
| **Propensity score matched cohorts** | 1110 | Continued ACEI/ARB | 1 |
|  |  | Stopped ACEI/ARB | 0.69 (0.42-1.14) |
| **Prescribing ACEI/ARB defined at 60 days after AKI discharge** | 1734 | Continued ACEI/ARB | 1 |
|  |  | Stopped ACEI/ARB | 0.92 (0.59-1.43) |
| **Cohort defined by 2 ACEI/ARB prescriptions in 60 days prior to AKI admission, 1 within 30 days** | - | Continued ACEI/ARB | N/A (not available) |
|  |  | Stopped ACEI/ARB |  |
| **Outcome events defined from first diagnostic position only** | 1844 | Continued ACEI/ARB | 1 |
|  |  | Stopped ACEI/ARB | 0.76 (0.45-1.28) |
| **Excluding people who required dialysis during admission** | - | Continued ACEI/ARB | N/A (not available) |
|  |  | Stopped ACEI/ARB |  |
| **Stratified time intervals: 0-29 days after start of follow up** | 1844 | Continued ACEI/ARB | 1 |
|  |  | Stopped ACEI/ARB | 0.58 (0.23-1.49) |
| **Stratified time intervals: 30-89 days after start of follow up** |  | Continued ACEI/ARB | 1 |
|  |  | Stopped ACEI/ARB | 1.36 (0.54-3.46) |
| **Stratified time intervals: ≥90 days after start of follow up** |  | Continued ACEI/ARB | 1 |
|  |  | Stopped ACEI/ARB | 0.74 (0.44-1.25) |
| **Excluding people who were readmitted in the 30 days after discharge from baseline AKI admission** | 1445 | Continued ACEI/ARB | 1 |
|  |  | Stopped ACEI/ARB | 0.74 (0.46-1.19) |
| **English analyses** | | | |
| **Main Analysis** | 6629 | Continued ACEI/ARB | 1 |
|  |  | Stopped ACEI/ARB | 0.90 (0.77-1.05) |
| **Propensity score matched cohorts** | 5715 | Continued ACEI/ARB | 1 |
|  |  | Stopped ACEI/ARB | 0.92 (0.77-1.08) |
| **Prescribing ACEI/ARB defined at 60 days after AKI discharge** | 6151 | Continued ACEI/ARB | 1 |
|  |  | Stopped ACEI/ARB | 0.91 (0.76-1.08) |
| **Cohort defined by 2 ACEI/ARB prescriptions in 60 days prior to AKI admission, 1 within 30 days** | 4086 | Continued ACEI/ARB | 1 |
|  |  | Stopped ACEI/ARB | 0.94 (0.78-1.14) |
| **Outcome events defined from first diagnostic position only in HES** | 6629 | Continued ACEI/ARB | 1 |
|  |  | Stopped ACEI/ARB | 0.81 (0.63-1.03) |
| **Excluding people who required dialysis during admission** | 6391 | Continued ACEI/ARB | 1 |
|  |  | Stopped ACEI/ARB | 0.90 (0.77-1.06) |
| **Stratified time intervals: 0-29 days after start of follow up** | 6629 | Continued ACEI/ARB | 1 |
|  |  | Stopped ACEI/ARB | 0.86 (0.58-1.27) |
| **Stratified time intervals: 30-89 days after start of follow up** |  | Continued ACEI/ARB | 1 |
|  |  | Stopped ACEI/ARB | 0.88 (0.62-1.24) |
| **Stratified time intervals: ≥90 days after start of follow up** |  | Continued ACEI/ARB | 1 |
|  |  | Stopped ACEI/ARB | 0.91 (0.76-1.11) |
| **Excluding people who were readmitted in the 30 days after discharge from baseline AKI admission** | 5127 | Continued ACEI/ARB | 1 |
|  |  | Stopped ACEI/ARB | 0.85 (0.71-1.02) |

**Table S8.** Summary of main results in English and Swedish cohorts and all sensitivity analyses for AKI as an outcome comparing people who stopped or continued ACEI/ARB after admission with AKI

|  | **Number included** | **Exposure** | **Adjusted hazard ratio**  **for stroke**  **(95% CI)** |
| --- | --- | --- | --- |
| **Swedish analyses** | | | |
| **Main Analysis** | 1894 | Continued ACEI/ARB | 1 |
|  |  | Stopped ACEI/ARB | 0.56 (0.34-0.93) |
| **Propensity score matched cohorts** | 1146 | Continued ACEI/ARB | 1 |
|  |  | Stopped ACEI/ARB | 0.48 (0.25-0.92) |
| **Prescribing ACEI/ARB defined at 60 days after AKI discharge** | 1789 | Continued ACEI/ARB | 1 |
|  |  | Stopped ACEI/ARB | 0.83 (0.49-1.40) |
| **Cohort defined by 2 ACEI/ARB prescriptions in 60 days prior to AKI admission, 1 within 30 days** | - | Continued ACEI/ARB | N/A (not available) |
|  |  | Stopped ACEI/ARB |  |
| **Outcome events defined from first diagnostic position only** | 1894 | Continued ACEI/ARB | 1 |
|  |  | Stopped ACEI/ARB | 0.52 (0.32-0.87) |
| **Excluding people who required dialysis during admission** | - | Continued ACEI/ARB | N/A (not available) |
|  |  | Stopped ACEI/ARB |  |
| **Stratified time intervals: 0-29 days after start of follow up** | 1894 | Continued ACEI/ARB | 1 |
|  |  | Stopped ACEI/ARB | 0.46 (0.09-2.32) |
| **Stratified time intervals: 30-89 days after start of follow up** |  | Continued ACEI/ARB | 1 |
|  |  | Stopped ACEI/ARB | 0.60 (0.16-2.26) |
| **Stratified time intervals: ≥90 days after start of follow up** |  | Continued ACEI/ARB | 1 |
|  |  | Stopped ACEI/ARB | 0.57 (0.32-1.00) |
| **Excluding people who were readmitted in the 30 days after discharge from baseline AKI admission** | 1445 | Continued ACEI/ARB | 1 |
|  |  | Stopped ACEI/ARB | 0.46 (0.25-0.85) |
| **English analyses** | | | |
| **Main Analysis** | 6869 | Continued ACEI/ARB | 1 |
|  |  | Stopped ACEI/ARB | 0.99 (0.71-1.38) |
| **Propensity score matched cohorts** | 5941 | Continued ACEI/ARB | 1 |
|  |  | Stopped ACEI/ARB | 0.90 (0.64-1.28) |
| **Prescribing ACEI/ARB defined at 60 days after AKI discharge** | 6443 | Continued ACEI/ARB | 1 |
|  |  | Stopped ACEI/ARB | 0.97 (0.68-1.37) |
| **Cohort defined by 2 ACEI/ARB prescriptions in 60 days prior to AKI admission, 1 within 30 days** | 4214 | Continued ACEI/ARB | 1 |
|  |  | Stopped ACEI/ARB | 0.96 (0.64-1.44) |
| **Outcome events defined from first diagnostic position only in HES** | 6869 | Continued ACEI/ARB | 1 |
|  |  | Stopped ACEI/ARB | 0.95 (0.67-1.33) |
| **Excluding people who required dialysis during admission** | 6617 | Continued ACEI/ARB | 1 |
|  |  | Stopped ACEI/ARB | 0.98 (0.70-1.37) |
| **Stratified time intervals** | - | Continued ACEI/ARB | N/A (too few events) |
|  |  | Stopped ACEI/ARB |  |
| **Excluding people who were readmitted in the 30 days after discharge from baseline AKI admission** | 5127 | Continued ACEI/ARB | 1 |
|  |  | Stopped ACEI/ARB | 0.84 (0.56-1.25) |

**Table S9.** Summary of main results in English and Swedish cohorts and all sensitivity analyses for stroke as an outcome comparing people who stopped or continued ACEI/ARB after admission with AKI

|  | **Number included** | **Exposure** | **Adjusted hazard ratio**  **for death**  **(95% CI)** |
| --- | --- | --- | --- |
| **Swedish analyses** | | | |
| **Main Analysis** | 1903 | Continued ACEI/ARB | 1 |
|  |  | Stopped ACEI/ARB | 0.94 (0.78-1.13) |
| **Propensity score matched cohorts** | 1162 | Continued ACEI/ARB | 1 |
|  |  | Stopped ACEI/ARB | 1.00 (0.81-1.24) |
| **Prescribing ACEI/ARB defined at 60 days after AKI discharge** | 1800 | Continued ACEI/ARB | 1 |
|  |  | Stopped ACEI/ARB | 1.04 (0.86-1.25) |
| **Cohort defined by 2 ACEI/ARB prescriptions in 60 days prior to AKI admission, 1 within 30 days** | - | Continued ACEI/ARB | N/A (not available) |
|  |  | Stopped ACEI/ARB |  |
| **Excluding people who required dialysis during admission** | - | Continued ACEI/ARB | N/A (not available) |
|  |  | Stopped ACEI/ARB |  |
| **Stratified time intervals: 0-29 days after start of follow up** | 1903 | Continued ACEI/ARB | 1 |
|  |  | Stopped ACEI/ARB | 0.98 (0.58-1.64) |
| **Stratified time intervals: 30-89 days after start of follow up** |  | Continued ACEI/ARB | 1 |
|  |  | Stopped ACEI/ARB | 1.38 (0.86-2.22) |
| **Stratified time intervals: ≥90 days after start of follow up** |  | Continued ACEI/ARB | 1 |
|  |  | Stopped ACEI/ARB | 0.86 (0.69-1.07) |
| **Excluding people who were readmitted in the 30 days after discharge from baseline AKI admission** | 1445 | Continued ACEI/ARB | 1 |
|  |  | Stopped ACEI/ARB | 0.83 (0.66-1.05) |
| **English analyses** | | | |
| **Main Analysis** | 6895 | Continued ACEI/ARB | 1 |
|  |  | Stopped ACEI/ARB | 1.27 (1.15-1.41) |
| **Propensity score matched cohorts** | 5937 | Continued ACEI/ARB | 1 |
|  |  | Stopped ACEI/ARB | 1.30 (1.16-1.45) |
| **Prescribing ACEI/ARB defined at 60 days after AKI discharge** | 6474 | Continued ACEI/ARB | 1 |
|  |  | Stopped ACEI/ARB | 1.43 (1.29-1.59) |
| **Cohort defined by 2 ACEI/ARB prescriptions in 60 days prior to AKI admission, 1 within 30 days** | 4234 | Continued ACEI/ARB | 1 |
|  |  | Stopped ACEI/ARB | 1.27 (1.13-1.44) |
| **Outcome events defined from first diagnostic position only in HES** | - | Continued ACEI/ARB | N/A |
|  |  | Stopped ACEI/ARB |  |
| **Excluding people who required dialysis during admission** | 6642 | Continued ACEI/ARB | 1 |
|  |  | Stopped ACEI/ARB | 1.26 (1.14-1.39) |
| **Stratified time intervals: 0-29 days after start of follow up** | 6895 | Continued ACEI/ARB | 1 |
|  |  | Stopped ACEI/ARB | 1.59 (1.21-2.08) |
| **Stratified time intervals: 30-89 days after start of follow up** |  | Continued ACEI/ARB | 1 |
|  |  | Stopped ACEI/ARB | 1.41 (1.12-1.76) |
| **Stratified time intervals: ≥90 days after start of follow up** |  | Continued ACEI/ARB | 1 |
|  |  | Stopped ACEI/ARB | 1.18 (1.05-1.33) |
| **Excluding people who were readmitted in the 30 days after discharge from baseline AKI admission** | 5127 | Continued ACEI/ARB | 1 |
|  |  | Stopped ACEI/ARB | 1.16 (1.03-1.30) |

**Table S10.** Summary of main results in English and Swedish cohorts and all sensitivity analyses for mortality as an outcome comparing people who stopped or continued ACEI/ARB after admission with AKI


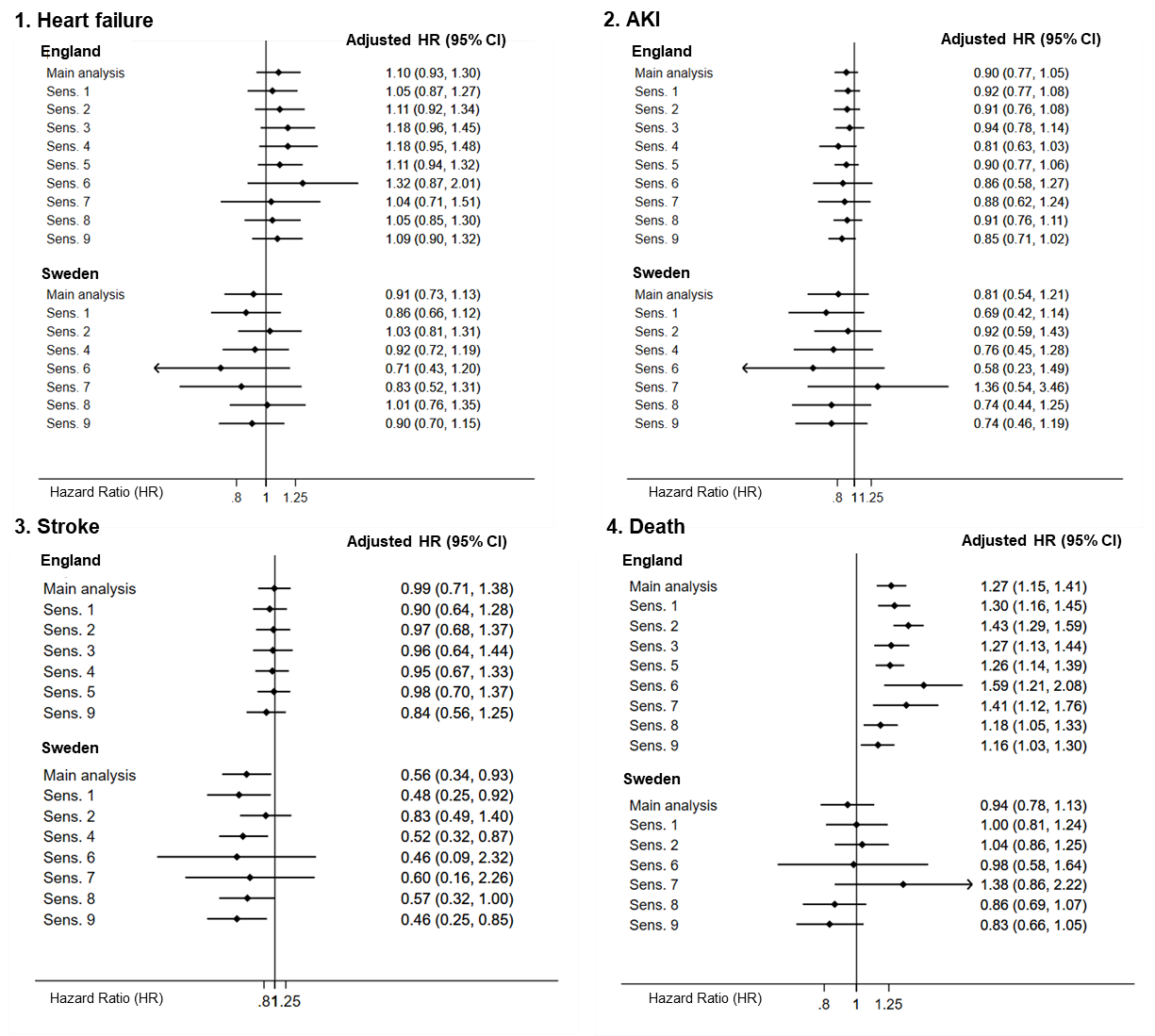


**Figure S5.** Summary of all analyses in English and Swedish cohorts for the study outcomes comparing people who stopped or continued ACEI/ARB after admission with AKI

| Sens. 1 | Propensity score matched cohort |
| --- | --- |
| Sens. 2 | Prescribing ACEI/ARB defined at 60 days after AKI discharge |
| Sens. 3 | Cohort defined by 2 ACEI/ARB prescriptions in 60 days prior to AKI admission, 1 within 30 days |
| Sens. 4 | Outcome events defined from first diagnostic position only |
| Sens. 5 | Excluding people who required dialysis during admission |
| Sens. 6 | Stratified time intervals: 0-29 days after start of follow-up |
| Sens. 7 | Stratified time intervals: 30-89 days after start of follow-up |
| Sens. 8 | Stratified time intervals: ≥90 days after start of follow-up |
| Sens. 9 | Excluding people who were readmitted in the 30 days after discharge from baseline AKI admission |
